# Astrocyte‐ and NMDA receptor‐dependent slow inward currents differently contribute to synaptic plasticity in an age‐dependent manner in mouse and human neocortex

**DOI:** 10.1111/acel.13939

**Published:** 2023-07-25

**Authors:** Andrea Csemer, Adrienn Kovács, Baneen Maamrah, Krisztina Pocsai, Kristóf Korpás, Álmos Klekner, Péter Szücs, Péter P. Nánási, Balázs Pál

**Affiliations:** ^1^ Department of Physiology, Faculty of Medicine University of Debrecen Debrecen Hungary; ^2^ Doctoral School of Molecular Medicine University of Debrecen Debrecen Hungary; ^3^ Department of Neurosurgery, Clinical Centre University of Debrecen Debrecen Hungary; ^4^ Department of Anatomy, Histology and Embryology, Faculty of Medicine University of Debrecen Debrecen Hungary; ^5^ Department of Dental Physiology and Pharmacology, Faculty of Dentistry University of Debrecen Debrecen Hungary

**Keywords:** aging, astrocyte, human brain, neocortex, NMDA receptor, pyramidal cell, slow inward current, synaptic plasticity

## Abstract

Slow inward currents (SICs) are known as excitatory events of neurons elicited by astrocytic glutamate via activation of extrasynaptic NMDA receptors. By using slice electrophysiology, we tried to provide evidence that SICs can elicit synaptic plasticity. Age dependence of SICs and their impact on synaptic plasticity was also investigated in both on murine and human cortical slices. It was found that SICs can induce a moderate synaptic plasticity, with features similar to spike timing‐dependent plasticity. Overall SIC activity showed a clear decline with aging in humans and completely disappeared above a cutoff age. In conclusion, while SICs contribute to a form of astrocyte‐dependent synaptic plasticity both in mice and humans, this plasticity is differentially affected by aging. Thus, SICs are likely to play an important role in age‐dependent physiological and pathological alterations of synaptic plasticity.

## Graphical abstract

Slow inward currents are consequences of astrocytic glutamate release (1) and neuronal GluN2B‐containing NMDA receptor activation (2) both in mice (left) and human (right; see symbols). Murine and human SICs differ in amplitude and kinetic parameters (3). In young and adult mice timing‐dependent synaptic depression or potentiation occurs; in humans, only potentiation was seen. With aging, synaptic plasticity by SICs is reduced in both species.

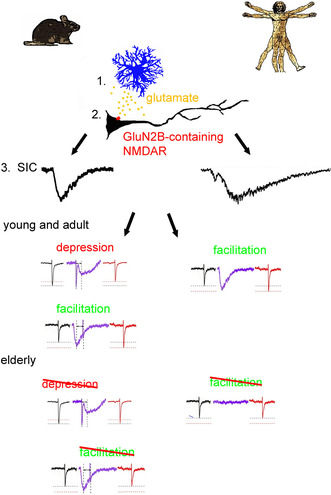

## 1 INTRODUCTION

It has been extensively demonstrated that astrocytes play multiple roles in synaptic plasticity, learning, and memory (Akther & Hirase, 2022). The NMDA receptor‐dependent long‐term potentiation (LTP) and short‐term memory were enhanced by astrocytic activation (Adamsky et al., 2018), whereas the latter one was impaired by inhibition of astrocytic Gq‐dependent signaling (Nagai et al., 2021). Astrocytic regulation of extrasynaptic glutamate homeostasis also contributes to the learning processes since astrocytic glutamate sets the threshold for LTP and blockade of glutamate uptake impairs spatial memory (Bechtholt‐Gompf et al., 2010; Bonansco et al., 2011).

Slow inward currents (SICs) of neurons were first demonstrated on hippocampal neuron‐astrocyte co‐cultures two decades ago (Araque et al., 1998). Since then, it was confirmed that SICs are NMDA‐receptor mediated cation currents with a significantly slower kinetics than excitatory postsynaptic currents (Bardoni et al., 2010; Fellin et al., 2004; Reyes‐Haro et al., 2010). Glutamate release eliciting these events is the consequence of astrocytic activity (Angulo et al., 2004; Chen et al., 2012; Fellin et al., 2004; Perea et al., 2014). SICs are present in several areas of the central nervous system (CNS) including the spinal cord, brainstem nuclei, as well as diencephalic and forebrain structures (Bardoni et al., 2010; Chen et al., 2012; Perea et al., 2014; Pirttimaki & Parri, 2012; Reyes‐Haro et al., 2010).

Despite of the extensive description of the phenomenon, little is known about the functions of SICs. These events occur simultaneously on neighboring neurons in rostral brain structures from the diencephalon to the neocortex. Based on these findings, it was hypothesized that SICs synchronize neuronal somata within the same astrocytic domain (Angulo et al., 2004; D'Ascenso et al., 2007; Fellin et al., 2004; Pirttimaki et al., 2011). Interestingly, similar synchronized occurrence has not been described on brainstem neurons (Reyes‐Haro et al., 2010).

Modeling and experimental studies rose the possibility that SICs have a modulatory role in setting synaptic strength, especially by regulating long‐term potentiation threshold and spike timing‐dependent plasticity (STDP; Bonansco et al., 2011; De Pittà & Brunel, 2016; De Pittà et al., 2011; Wade et al., 2011). However, this possibility has never been confirmed by experiments under in vivo circumstances.

Human astrocytes are not only far less studied than their rodent counterparts, but also show crucial differences in astrocytic morphology, subtypes, and functional properties. The astrocyte‐neuron ratio is also different between rodents and humans, as astrocytes outnumber neurons in humans (Oberheim et al., 2009, 2012). Furthermore, implantation of human astrocytes to the murine brain improved learning performance of mice (Han et al., 2013) indicating a significantly stronger contribution of human astrocytes to cognitive functions.

Regarding electrical astrocyte‐neuron communication, there are basic similarities between rodents and humans; as the presence of SICs as astrocyte‐ and NMDA receptor‐dependent excitatory events on neurons were demonstrated in the human cortex (Navarrete et al., 2013).

Astrocytes are affected by aging. Beyond morphological alterations of the astrocytic domain and arborization (Popov et al., 2021; Robillard et al., 2016), functional changes have also been reported. The spread of calcium signals and decline of astrocytic responsiveness were detected together with changes in the electrical membrane properties (Popov et al., 2021). Aging also increases the probability of A1‐like (reactive neuroinflammatory) reactivity of astrocytes and decreases the capacity for glutamate clearance supporting excitotoxicity (Boisvert et al., 2018; Clarke et al., 2018; Sakurai et al., 2022).

As neuronal functions in general, the glutamatergic astrocyte‐neuron communication also declines with age in rodents. Astrocytic glutamate clearance, its responsiveness to mGluR5 activation and the amplitude of the excitatory postsynaptic currents (EPSCs) decreases as well, while the extracellular glutamate concentration increases with age. However, no change was observed in astrocytic calcium waves and NMDA receptor‐dependent neuronal responses to astrocyte activation (Gómez‐Gonzalo et al., 2017). Regulation of STDP in mice also changes with age (Martínez‐Gallego et al., 2022).

Our aim was to assess SICs' contribution to synaptic plasticity, together with or independent from any other parallel astrocytic actions on neurons. We also aimed to investigate the age dependence of SICs and putative interspecies differences in the above actions between rodents and humans.

It was found that SICs are present in all ages in mice with a moderate decline with age. SICs are capable of eliciting spike timing‐dependent plasticity (STDP) in mice. In humans, SICs had slower kinetics and greater charge transfer than in mice. The age dependence of SICs in humans is stronger as could not be detected in samples from patients above the age of 70. Human SICs also seemed to contribute to synaptic plasticity.

## 2 MATERIALS AND METHODS

### 2.1 Solutions, chemicals

In all patch clamp and calcium imaging experiments, artificial cerebrospinal fluid (aCSF) was used as a general buffer solution for the preparations. The aCSF was composed of the following material (in mM): NaCl, 120; KCl, 2.5; NaHCO_3_, 26; glucose, 10; NaH_2_PO_4_, 1.25; myo‐inositol, 3; ascorbic acid, 0.5; sodium‐pyruvate, 2; CaCl_2_, 2; MgCl_2_, 1; pH 7.4. When SICs were recorded, magnesium was omitted from normal aCSF (naCSF) to maintain nominally magnesium‐free medium for the tissue. Brain slices were prepared in low Na^+^ aCSF, where 95 mM NaCl was replaced by glycerol (60 mM) plus sucrose (130 mM). All chemicals were purchased from Sigma‐Aldrich, unless otherwise stated.

### 2.2 Human samples

Collection and use of human tissue was performed in accordance with national and international guidelines. Protocols for sample collection and experiments were approved by the Hungarian National Public Health and Medical Officer Center (DE KK RKEB/IKEB 4259‐2014).

Samples from 13 patients between 38 and 74 years of age, from both genders (6 women and 7 men) were included. All patients had malignancy affecting neocortex. Five patients had glioblastoma multiforme, whereas 8 had carcinoma metastases. Intact neocortical tissues, removed during the approach of the tumor or for prevention of herniation, were used for slice preparation. Samples where any signs of tumor infiltration were seen in the sample were excluded from analysis (e.g., tumor cells or lymphocytes or reduction of neuronal action potential amplitude or firing rate were seen; Table 1; Figure S5). The samples were collected in the operating theater and transferred to the Department of Physiology in a chamber developed for this purpose. The chamber was filled with oxygenated ice‐cold low Na^+^ aCSF and was transported to the laboratory within 10 min after removal. In the laboratory, the tissue was sliced to 200 μm thick pieces perpendicularly to the brain surface (see below). The slicing and recording procedures applied for the human and murine samples were identical.

**TABLE 1:** Patient data.

Number	Age (years)	Gender	Location	Diagnosis
1	38	♀	Right parietal	Glioblastoma multiforme
2	45	♀	Right temporal cortex	Glioblastoma multiforme
3	59	♀	Right occipital	Carcinoma metastasis (breast cancer)
4	59	♂	Left frontal	Carcinoma metastasis (prostate cancer)
5	59	♂	Parietal	Glioblastoma multiforme
6	61	♀	Right frontal	Carcinoma metastasis (small cell neuroendocrine lung cancer)
7	63	♀	Right frontal	Carcinoma metastasis (lung cancer)
8	66	♂	Right temporoparietal	Glioblastoma multiforme
9	67	♂	Right temporal	Carcinoma metastasis (breast cancer)
10	68	♀	Temporal	Glioblastoma multiforme
11	69	♂	Left frontal	Glioblastoma multiforme
12	70	♂	Right frontal	Carcinoma metastasis (stomach cancer)
13	74	♂	Right frontal	Carcinoma metastasis (lung cancer)

### 2.3 Animals, preparation

Animal experiments were performed in accordance with the appropriate national and international (EU Directive 2010/63/EU for animal experiments) and institutional guidelines and laws on the care of research animals (5/2015/DEMÁB; 19/2019/DEMÁB). 9‐ to 585‐day‐old mice expressing tdTomato fluorescent protein in a glial fibrillary acidic protein (GFAP) dependent way (*n* = 12) or control mice (lox‐tdTomato; *n* = 71) from both sexes were used as experimental animals. For having GFAP–tdTomato expressed mice, homozygous floxed‐stop‐ tdTomato (B6;129S6‐Gt(ROSA)26Sor^tm9(CAG‐tdTomato)Hze/^J; Jax mice accession number: 007905) and GFAP‐cre (B6.Cg‐Tg(Gfap‐cre)73.12Mvs/J; Jax number: 012886) strains purchased from Jackson Laboratories were crossed in our animal facility.

The slices (coronal plane in the level of the parietal cortex with 200 μm thickness) were prepared in ice‐cold (cca. 0 to −2°C) low Na^+^ aCSF with a Microm HM 650 V vibratome (Microm International GmbH). The slices were incubated in normal aCSF for 1 h at 37°C prior to starting the experiment.

Young adult (9–12 weeks old) lox‐tdTomato mice from both sexes were subjected to stereotaxic injection of ready‐to‐use viruses carrying plasmids encoding hM3D(Gq) chemogenetic actuator and mCherry tag expressed under GFAP promoter (pAAV‐GFAP‐hM3D(Gq)‐mCherry (AAV5); a gift from Bryan Roth; Addgene plasmid # 50478; http://n2t.net/addgene:50478; RRID:Addgene_50478, *n* = 13; titer: 2 × 10^13^ GC/mL) or solely mCherry tag as control (pAAV‐GFAP104‐mCherry (AAV5), a gift from Edward Boyden; Addgene plasmid # 58909; http://n2t.net/addgene:58909; RRID:Addgene_58909, Perea et al., 2014, *n* = 10; titer: 1.7 × 10^13^ GC/mL). Mice were anesthetized with intraperitoneal injection of ketamine (100 mg/kg) and xylazine (10 mg/kg) and heated to 37°C during surgery with a modified plate heater (Linkam CO102; Linkam Scientific Instruments Ltd.). Drying of eyes was prevented by placing drops of physiological saline on the eyes. The head of the anesthetized mouse was fixed in a stereotaxic frame (RWD Life Science Co., LTD). After opening the skin, the skull was unilaterally trepanated with a microdrill (RWD Life Science Co., LTD) 1.5 mm from midline, 1 mm caudally from bregma. 100–100 nL of the viruses specified above was injected to the cortical surface and also into 200 and 400 μm depth of the parietal cortex with a Hamilton syringe and microinjector (RWD Life Science Co., LTD). After operation, the skull was covered with bone wax and the skin was sutured (5‐0 Vicryl, Ethicon). After the anesthesia wore off and during the next 2 postoperative days, mice received ibuprofen for pain relief (30 mg/kg; Nurofen Baby, Reckitt Benckiser Ltd.). These mice were kept in individually ventilated cages for 7–10 days following surgery. After this time, mice were sacrificed and brain slices were prepared as detailed above.

### 2.4 Electrophysiology

Neocortical pyramidal neurons in layer III‐IV were patched. The resistance of the patch pipettes was 6–8 MΩ, and the internal solution contained (in mM): K‐gluconate, 120; NaCl, 5; 4‐(2‐hydroxyethyl)‐1‐ piperazineethanesulfonic acid (HEPES), 10; Na_2_‐ phosphocreatinine, 10; ethylene glycol‐bis(β‐aminoethyl ether)‐N,N,N′,N′‐tetraacetic acid (EGTA), 2; CaCl_2_, 0.1; Mg‐ATP, 5; Na_3_‐GTP, 0.3; biocytin, 8; pH 7.3. Whole cell patch clamp experiments were performed at room temperature (24–26°C) with an Axopatch 200A amplifier (Molecular Devices) on randomly chosen neocortical pyramidal neurons (Figure S5C,D). All data were recorded with Clampex 10.0 software (Molecular Devices), and Clampfit 10.0 (Molecular Devices) Synaptosoft MiniAnalysis (Synaptosoft) softwares were used for data analysis. Both voltage and current clamp configurations were used with below 20 MΩ series resistance, with less than 10% change were included and only stable recordings with minimal leak currents were considered.

In current clamp experiments, 1‐s‐long square current pulses, ranging from −30 pA to +120 pA with 10 pA increments, were applied on human pyramidal neurons to check neuronal viability and exclude neurons where tumor infiltration might affect their excitability.

Gap‐free recordings for detecting SICs and sEPSCs were performed under voltage‐clamp configuration, from −60 mV holding potential. Evoked EPSCs were also recorded in voltage clamp configuration. In these experiments, the presynaptic fibers innervating the studied neuron were stimulated with a tungsten bipolar electrode and inserted 50–100 μm away from the soma. The stimulation electrode was connected to a BioStim STC‐7a stimulator (Supertech). The amplitude of the stimuli was set to a value where the failures practically disappeared. Single stimuli were delivered in every 20 s. Recordings were performed in naCSF and in nominally magnesium‐free aCSF. In some experiments testing the subunit composition of NMDA receptors, 500 nM PPPA (GluN2A‐specific inhibitor; Tocris Cookson Ltd., Feng et al., 2005; Peng et al., 2008; Takeda et al., 2012), 5 μM ifenprodil (GluN2B‐specific inhibitor, Tocris Cookson Ltd., Matta et al., 2011; Nuno‐Perez et al., 2021; Reynolds & Miller, 1989) and 10 μM D‐AP5 (nonspecific inhibitor, Tocris Cookson Ltd., Davies & Watkins, 1982) was administered. In other experiments, 10 and 100 μM WAY213613 (Tocris Cookson Ltd.) was used for selective inhibition of glutamate uptake via EAAT2 (Brancaccio et al., 2017; Dumont et al., 2014; Dunlop et al., 2005). The EAAT1‐specific inhibitor UCPH101 was administered in 10 and 25 μM (Tocris Cookson Ltd., Brancaccio et al., 2017; Dumont et al., 2014), and the nonspecific EAAT inhibitor DL‐TBOA was used in 100 μM (Jabaudon et al., 1999; Shimamoto et al., 1998).

Electrical stimulation was combined with chemogenetic activation of astrocytes. In these experiments, mice were injected with virus vectors carrying the plasmids described above. Control experiments were performed in naCSF and in 0.1% DMSO (since CNO stock solution was dissolved in DMSO) with additional strychnine (1 μM) and bicuculline (10 μM) to block inhibitory synaptic neurotransmission. After recording under control conditions, clozapine‐N‐oxyde (CNO, 10 μM; Tocris Cookson Ltd.) was administered. Only those recordings were considered where spontaneously occurring but chemogenetically provoked SICs developed within 1 s compared to the EPSC peak.

In another arrangement, glutamate was released by flash photolysis. In this case, 30 μM MNI‐caged glutamate (Tocris Cookson Ltd.) was applied in the bath chamber and the whole chamber was illuminated with UV and near‐UV light (with wavelength below 395 nm) by using a Rapp flash lamp (Rapp OptoElectronic GmbH).

In the third set of stimulation experiments, a previously recorded SIC of a murine neocortical pyramidal neuron was used as a command signal applied together with the presynaptic electrical stimulation. In the latter two experiments, the timing between the stimulation and the glutamate release or the artificial SIC was set to different values. At the end of all recordings of SICs or EPSCs, a cocktail of NBQX (10 μM), D‐AP5 (10 μM), strychnine (1 μM), and bicuculline (10 μM) was used to inhibit SIC activity and fast synaptic neurotransmission to exclude artifacts resulting from direct stimulation of the investigated neuron or from unexpected noise mimicking events.

Only those recordings were taken into account where the SIC (or glutamate current) charge transfer was greater than 1 pC—other recordings were excluded from the analysis.

Visualization of the genetically encoded fluorescent marker (tdTomato) was accomplished by using a fluorescent imaging system (Till Photonics GmbH) equipped with a xenon bulb‐based Polychrome V light source, a CCD camera (SensiCam, PCO AG), an imaging control unit (ICU), and the Till Vision software (version 4.0.1.3).

### 2.5 Morphological and immunohistochemical evaluation

Neurons from electrophysiological experiments were filled with biocytin, and samples were fixed (4% paraformaldehyde in 0.1 M phosphate buffer; *pH* 7.4; 4°C) for morphological identification of neurons. Permeabilization was achieved using Tris buffered saline (in mM, Tris base, 8; Trisma HCl, 42; NaCl, 150; *pH* 7.4) supplemented with 0.1% Triton X‐100 and 10% bovine serum (60 min). After this step, phosphate buffer containing streptavidin‐conjugated Alexa488 (1:300; Molecular Probes Inc.) was applied for 90 min.

Neurons and the mCherry tag expressed by astrocytes following virus injection were visualized under confocal microscope (Zeiss LSM 510; Carl Zeiss AG); tile scan images were taken with 40× objective and 1 μm z stacks.

Immunohistochemical studies were performed on 4% paraformaldehyde fixed brain slices of young (8–30 days old), adult (90–200 days old), and old mice (older than 1 year), as well as on samples from middle‐aged and old human patients (38 and 69 years old). The fixed samples were washed (3 × 10 min) in Tris buffered saline (TBS), and 50 μm thick slices were prepared using a vibrating microtome (Campden Instruments). Double immunofluorescence labeling was performed on free‐floating slices. Blocking and permeabilization steps were applied using TBS supplemented with 0.1% Triton X‐100 and 10% normal donkey serum for 1 h at room temperature. Samples were incubated with the primary antibodies (rabbit anti‐Glun2B [Cell Signaling Technology, 1:100], mouse anti‐GFAP [Synaptic Systems, 1:1000], mouse anti‐synaptophysin [Sigma‐Aldrich, 1:200].) diluted in TBS containing 1% of normal donkey serum and 0.1% Triton X‐100 (48 h at 4°C).

The samples were then washed three times with TBS and incubated with fluorescent secondary antibodies (goat anti‐rabbit Cy3 [Life Technologies, 1:1000], donkey anti‐mouse Alexa 488 [Sigma‐Aldrich, 1:1000]) and Nissl staining (Molecular Probes, 1:200). Nissl staining was performed on the samples in parallel with the secondary antibodies. Finally, the samples were covered using a DAPI (4′,6‐diamidino‐2‐phenylindole) containing mounting medium (Vector Laboratories).

The fluorescent images were taken using Zeiss laser scanning confocal microscope (Zeiss LSM880 Airyscan; Zeiss). At least three images were taken from each immunohistochemical staining. We selected three ROIs in each image, and the mean fluorescence intensity (MFI) was measured using the ImageJ program (National Institute of Health).

All data represented as mean ± SEM. Normality tests are used to determine the normal distribution of datasets. Statistical comparison of two datasets was assessed with two sample Student's *t* test, whereas multiple comparisons were performed using Tukey's multiple comparisons test. Changes were considered significant when *p* < 0.05. Pearson's correlation coefficient (*r*) was applied to assess correlations between parameters by plotting them against each other and by the linear fit of the dataset.

## 3 RESULTS

### 3.1 Both murine and human SICs are astrocyte‐ and GluN2B subunit containing NMDA receptor‐dependent events

First, we confirmed that the events to be investigated are identical with the previously described SICs (Angulo et al., 2004; Chen et al., 2012; Fellin et al., 2004; Perea et al., 2014), as these are (a) clearly distinguishable from EPSCs on the basis of their kinetic properties, (b) mediated by GluN2B‐containing NMDA receptors, and (c) consequences of astrocyte activation.

**FIGURE 1:**
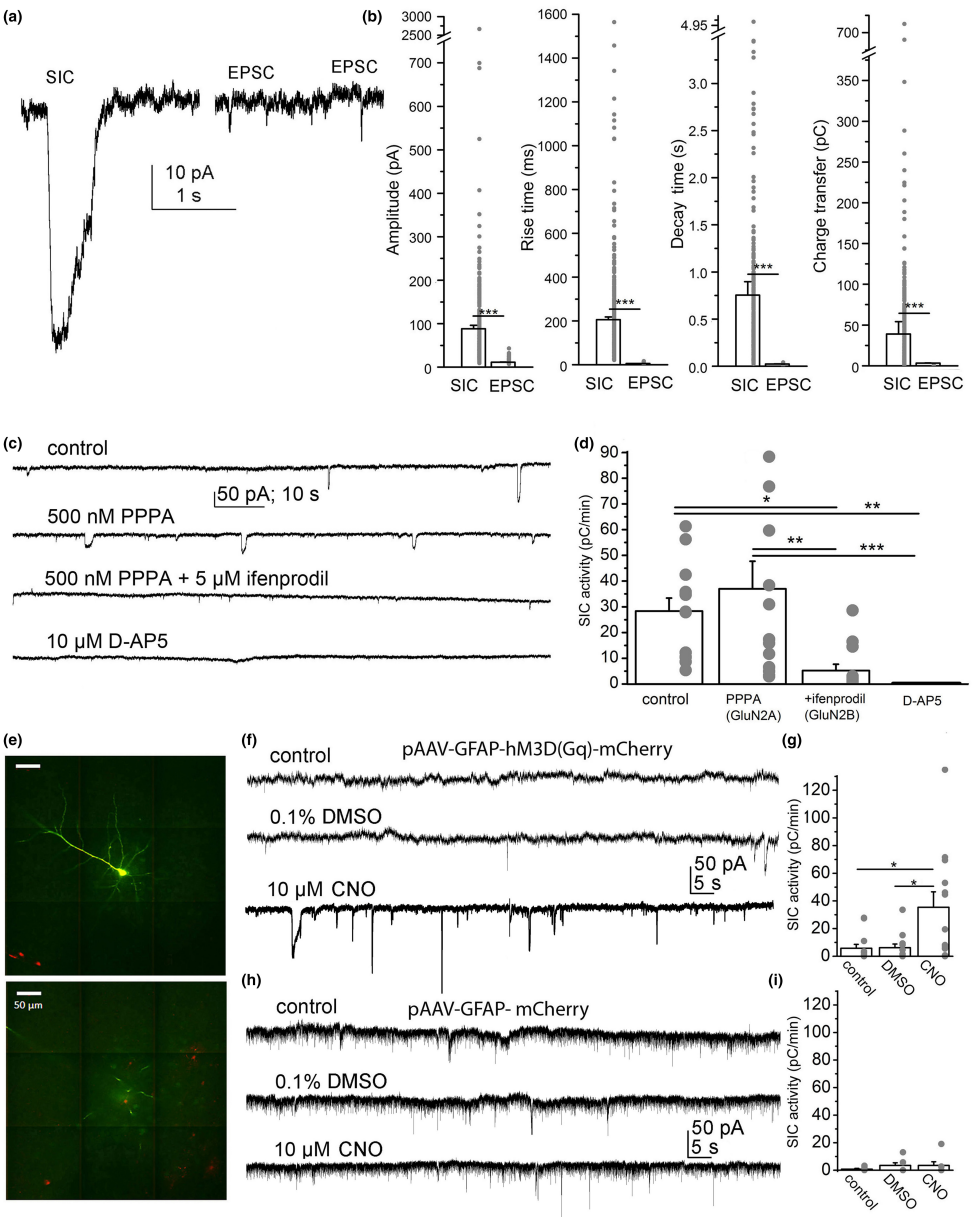
Slow inward currents (SICs) of murine neocortical pyramidal neurons are slow events mediated by activation of NMDA receptors and astrocytes. (a) The electrophysiological parameters of SICs unambiguously distinguish them from EPSCs. Representative records of a SIC (left) and two EPSCs (right). (b) Statistical comparison of SIC and EPSC amplitudes, rise and decay times and charge transfer data (“area”) of the individual events (hollow columns: average ± SEM, gray dots: individual data). (c) SICs are mediated by NMDA receptors with GluN2B subunits. Representative recordings under control conditions (0 Mg^2+^ naCSF), with 500 nM PPPA (GluN2A subunit specific NMDA receptor inhibitor), with additional ifenprodil (5 μM, GluN2B‐specific NMDA receptor blocker) and with 10 μM D‐AP5 (nonspecific NMDA receptor inhibitor). (d) Statistical comparison of charge transfer by SICs in a minute (“SIC activity”) in the presence of the drugs shown in panel ‘c’ (hollow columns: average ± SEM, gray dots: individual data). (e) A neocortical pyramidal cell labeled with biocytin during the patch clamp experiment (green) and astrocytes expressing mCherry tag (red) on two confocal z‐stack images (scale bar: 50 μm). (f) Representative traces under control conditions, with 0.1% DMSO and with 10 μM CNO from a sample expressing hM3D(Gq) chemogenetic actuator under GFAP promoter. (g) Statistical comparison of SIC activity under the conditions shown on panel ‘f’ (hollow columns: average ± SEM, gray dots: individual data). (h) Representative traces recorded in control, with DMSO, and 10 μM CNO from a sample lacking hM3D(Gq) chemogenetic actuator but expressing only mCherry tag under GFAP promoter. (i) Statistical comparison of SIC activity under conditions shown on panel ‘h’ (hollow columns: average ± SEM, gray dots: individual data). *p < 0.05; **p < 0.01; ***p < 0.001.

Pyramidal neurons of murine coronal neocortical samples were patched and gap‐free recordings were performed at −60 mV holding potential while the normal artificial cerebrospinal fluid (naCSF) was exchanged to nominally magnesium‐free aCSF. SICs were present in naCSF as well, but the magnesium‐free solution radically increased the SIC activity (i.e., the charge transfer by SICs per minute). As expected, this recording solution increased the EPSC amplitude and prolonged both the rise and decay time. As a consequence, the charge transfer by EPSCs was also increased (Figure S1A,B). All investigated parameters including the amplitude, rise and decay time and charge transfer by a single event (“area”) were significantly greater for SICs than EPSCs (Table S1). Datasets for SICs and EPSCs were separable by a cutoff value in case of the rise time (Figure 1a,b). For both murine and human neocortical SICs, this value was 20 ms.

The subunit composition of NMDA receptors responsible for SICs was also tested by application of PPPA (GluN2A subunit selective inhibitor), ifenprodil (GluN2B subunit selective blocker), and D‐AP5 (non‐selective NMDA receptor inhibitor). PPPA on its own did not affect SIC activity, in contrast to ifenprodil which largely reduced it. SIC activity was fully abolished by application of D‐AP5 (Table S2; Figure 1c,d).

Chemogenetic stimulation was used to activate astrocytes by expressing hM3D chemogenetic actuator (Figures S2 and S3). Compared to the aCSF and 0.1% DMSO controls (the stock CNO was dissolved in DMSO), activation with CNO significantly increased SIC activity. These changes were not observed if astrocytes expressed only the mCherry tag (Table S2, Figure 1e–i).

**FIGURE 2:**
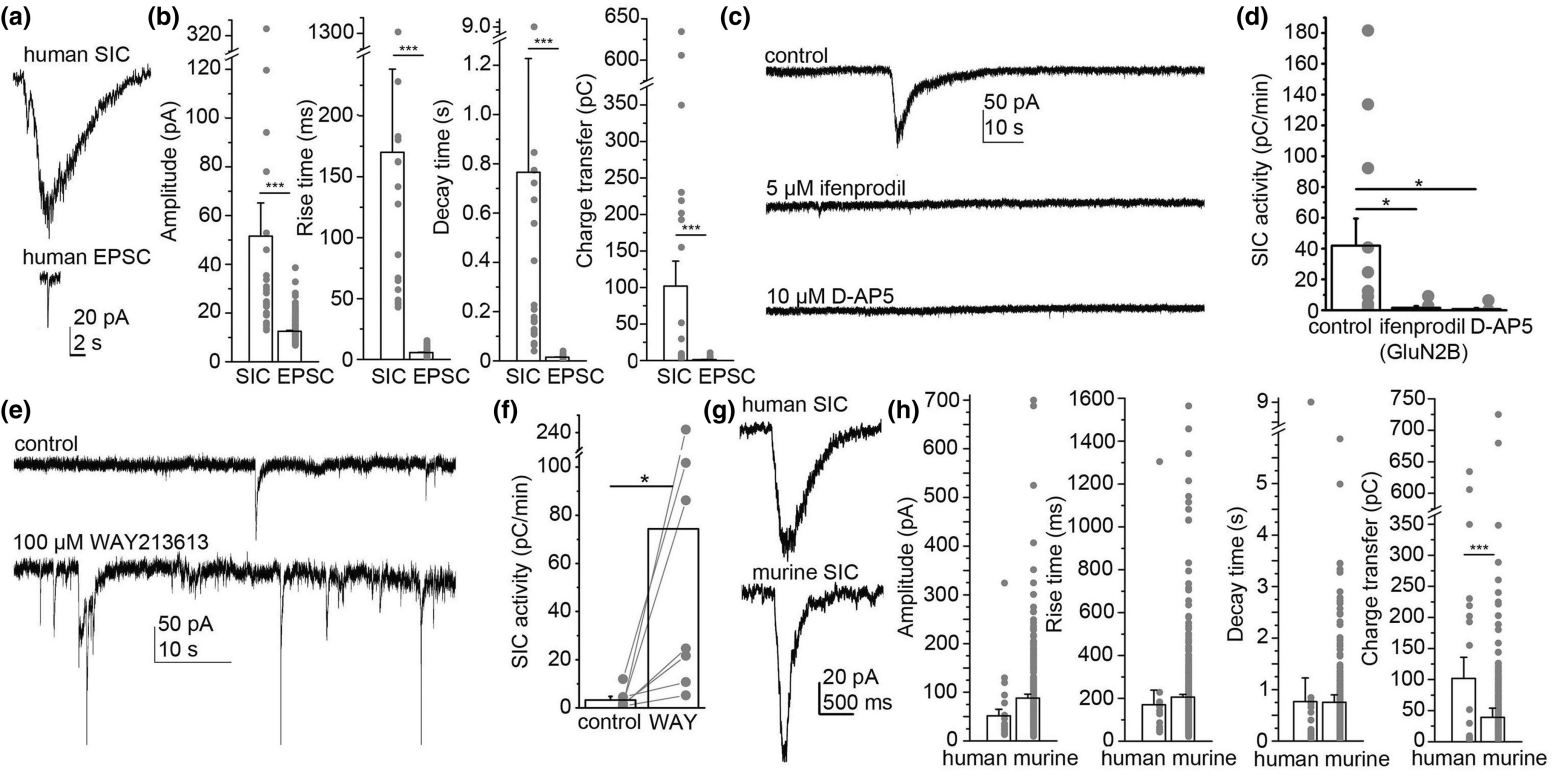
Human slow inward currents (SICs) are also NMDA receptor‐ and astrocyte‐dependent events with greater charge transfer than murine SICs. (a) Representative traces of a human SIC (above) and an EPSC (below). (b) Statistical comparison of human SIC and EPSC amplitudes, rise and decay times and charge transfer data (“area”) of the individual events (hollow columns: average ± SEM, gray dots: individual data). (c) SICs are mediated by GluN2B‐containing NMDA receptors in humans as well. Representative recordings in control (magnesium‐free naCSF), with ifenprodil (5 μM, GluN2B‐specific NMDA receptor blocker) and 10 μM D‐AP5 (nonspecific NMDA receptor inhibitor). (d) Statistical comparison of the charge transfer by SICs in a minute (“SIC activity”) in the presence of the drugs shown in panel ‘c’ (hollow columns: average ± SEM, gray dots: individual data). (e) Representative traces of SIC recordings taken under control conditions (above) and with 100 μM WAY213613 (EAAT2‐specific glutamate uptake inhibitor). (f) Statistical analysis of the SIC activity measured under the two conditions shown in panel ‘e’ (hollow columns: average ± SEM, gray dots and lines: individual data). (g) Representative traces of human (above) and murine SICs (below). (h) Statistical comparison of human and murine SIC parameters (hollow columns: average ± SEM, gray dots: individual data). Note that the charge transfer in human SICs is significantly greater than in mice. *p < 0.05; ***p < 0.001.

Similar observations were made when studying human neocortical samples. SIC activity and EPSC parameters were increased in magnesium‐free aCSF (Figure S1C,D and S5). SICs were significantly slower events than EPSCs (Table S1; Figure 2a,b), and SIC activity was fully abolished by ifenprodil or D‐AP5 (Table S2; Figure 2c,d). In the absence of chemo‐ or optogenetic activation of human astrocytes, we applied an EAAT2‐specific glutamate transport inhibitor WAY213613 to model the actions of astrocytic glutamate release on SICs which drastically increased SIC activity in the human samples (Table S2; Figure 2e,f). In order to test the actions of EAAT1 inhibition on appearance of SICs, the nonspecific DL‐TBOA was used on both human and murine samples. It was found that addition of TBOA to WAY213613 or application of it separately did not result in statistically different stimulation of SIC activity (Figure S4A,B). When UCPH101 was used on murine samples to specifically inhibit EAAT1, no increase in SIC activity was found (Figure S4A).

These results indicate that the events detected by us in murine and human samples are identical with those referred as SICs in the literature (Angulo et al., 2004; Chen et al., 2012; Fellin et al., 2004; Perea et al., 2014).

### 3.2 Human SICs are larger than murine ones

When individual human and murine SICs were compared, some differences could be revealed (Figure 2g,h). Although the differences were not significant, there was a tendency for the human SIC to have a smaller amplitude, which might be at least partially explained with the circumstances of tissue sample transfer (Table S1, Figure S6). Furthermore, the charge transfer was significantly greater in the human than murine SICs This parameter was not affected by the different tissue handling (Table S1).

When comparing middle‐aged populations, SIC amplitude was significantly smaller, whereas decay time and charge transfer were significantly greater than in mice (Figure S7). In conclusion, human SICs display slower kinetics, which—despite of the probably smaller amplitude—might explain the significantly greater charge transfer than in mice.

### 3.3 SICs work as electrical signals to elicit spike timing‐dependent plasticity

Since it is widely accepted that astrocytes can elicit or alter synaptic plasticity (Adamsky et al., 2018; Bonansco et al., 2011; Han et al., 2013; Martínez‐Gallego et al., 2022) and SICs can influence neuronal excitability (Angulo et al., 2004; Chen et al., 2012; Fellin et al., 2004; Perea et al., 2014). Therefore, we hypothesized that SICs might directly act on synaptic plasticity.

**FIGURE 3:**
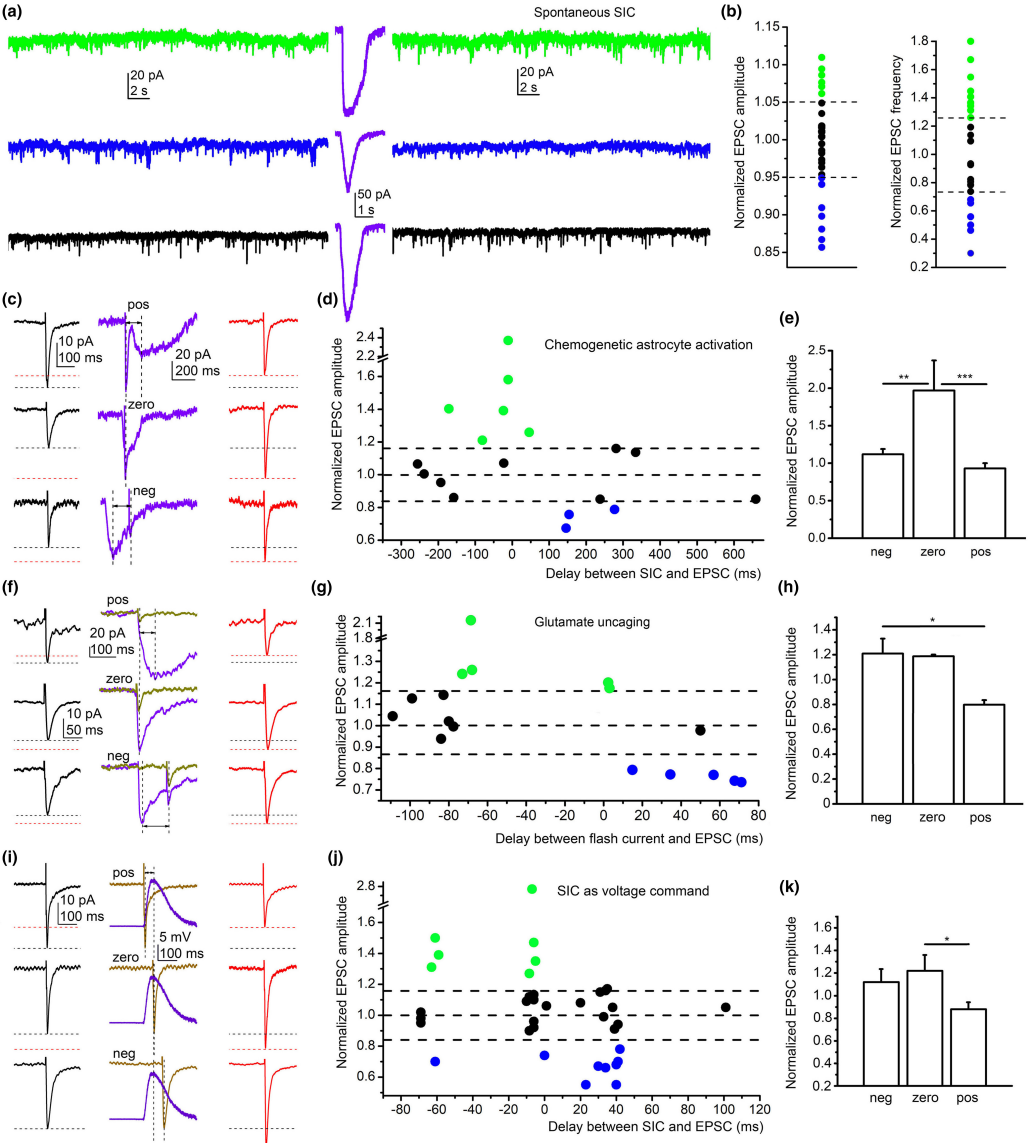
Slow inward currents (SICs) as electrical events are capable of eliciting synaptic plasticity in mice. (a, b) Changes of spontaneous EPSCs by spontaneously occurring SICs. (a) Representative gap‐free recordings from mouse pyramidal neurons. Left: gap‐free recordings prior to the first SIC. Purple traces: SICs. Right traces: gap‐free recordings following the first SIC. Green: Increased EPSC frequency and amplitude, blue: decreased EPSC frequency and amplitude, black: no change. (b) All data points of EPSC amplitudes and frequencies after the first SIC normalized to the data obtained prior to the SIC. Green dots: increase, blue dots: decrease, black dots: no change, dashed lines: limits of spontaneous fluctuations (5%). (c–e) Spike timing‐dependent changes of evoked EPSCs by SICs elicited with chemogenetic astrocyte activation by 10 μM CNO. (c) Representative EPSC and SIC traces. Black traces, left: averages of 10 EPSCs evoked prior to SICs; purple traces: evoked EPSCs and SICs with different timings indicated by dashed lines and arrows (pos: SIC follows the EPSC; zero: the EPSC and SIC peaks occur at the same time; neg: the SIC precedes the EPSC). Red traces, right: average of 10 EPSCs evoked after the SIC. Black dashed lines indicate the EPSC peaks prior to the SIC, the red dashed lines represent the amplitudes of EPSCs after the SIC. (d) Individual data points of EPSC amplitudes after the SIC, normalized to amplitudes before the SIC and plotted against the time differences measured between the EPSCs and SICs. Green: increase, blue: decrease, black: no change. Dashed lines indicate 100% and ± 16% as the limits of fluctuation. (e) Statistical comparison of EPSC amplitudes where the SIC precedes the EPSC (neg), occurs at the same time (zero) or follows it (pos). Columns: average ± SEM. (f–h) Spike timing‐dependent changes by SIC‐like events elicited by glutamate uncaging by using 30 μM MNI‐caged glutamate. (f) Representative EPSC and SIC traces. Black traces, left: averages of 10 EPSCs evoked prior to uncaging; dark yellow traces: evoked EPSCs, purple traces: glutamate currents elicited by uncaging with different timings indicated by dashed lines and arrows (pos: glutamate current follows the EPSC; zero: the EPSC and glutamate current peaks occur at the same time; neg: the glutamate current precedes the EPSC). Red traces, right: average of 10 evoked EPSCs following uncaging. (g) Individual data points of normalized EPSC amplitudes after uncaging plotted against the time differences between the EPSCs and glutamate currents using the same arrangement as in ‘d’. (h) Statistical comparison of EPSC amplitudes where uncaging precedes the EPSC (neg), occurs at the same time (zero) or follows it (pos). Columns: average ± SEM. (i–k) Spike timing‐dependent changes of evoked EPSCs by depolarization elicited by a previously recorded SIC used as voltage command with a similar arrangement as on panels ‘f–h’. *p < 0.05; **p < 0.01; ***p < 0.001.

First, we recorded spontaneous EPSC and SIC activity from the holding potential of −60 mV using a gap‐free protocol. Recordings were started in naCSF followed by magnesium‐free aCSF. Only those recordings were involved in our analysis where the EPSC parameters in the magnesium‐free solution became stable before SICs occurred (Figure S1B). Only recordings with SICs greater than 1 pF charge transfer were considered. Spontaneous EPSC (sEPSC) amplitudes and frequencies obtained before and after the appearance of the first SIC were compared. Both frequency and amplitude were increased in some cases (34.6% for frequency and 23% for amplitude), whereas a decrease was seen in other recordings (27% for both frequency and amplitude). However, many times no change was detected (38.4% for frequency and 50% for amplitude, *n* = 26). The EPSC amplitude and frequency were considered to be unaltered within 5% change; the range of changes found under control conditions without SICs (Figure 3a,b). Subsequently, SICs can alter EPSC activity indicating that a form of synaptic plasticity might be elicited by these events.

When SICs were elicited by increased ambient glutamate concentration (by inhibition of glutamate uptake with WAY213613, DL‐TBOA or UCPH101), EPSC amplitudes and frequencies were similarly altered as in case of spontaneously occurring SICs; no significant difference was seen between the actions of spontaneous SICs and the ones elicited by inhibition of glutamate uptake (Figure S8).

To further investigate this potential synaptic plasticity elicited by SICs, astrocytes expressing hM3D chemogenetic actuator were activated by bath application of CNO. Excitatory synaptic inputs of pyramidal neurons were electrically stimulated simultaneously. If a SIC was generated and developed within a 1 s time frame evoked EPSC peak, the change of EPSC amplitude was analyzed. In 50% of all cases, long lasting changes of EPSC amplitudes were seen (*n* = 18). If the SIC preceded or occurred at the same time as the evoked EPSC, 21%–137% increase in EPSC amplitude was obtained. If the SIC was seen after the evoked EPSC, 22%–33% decrease was detected (in 3 cases). These changes lasted for the entire 40 min of the recordings. In another 50% of the recordings, the change in EPSC amplitude was less than 16%, corresponding to the range of spontaneous fluctuation (Figure 3c–e). Taken all data together where SICs preceded EPSCs (“negative shift”), or occurred at the same time (“zero shift”) the EPSC amplitude increased, whereas if the SIC appeared after the EPSC (“positive shift”), the normalized amplitude decreased. On the basis of these results, it is concluded that chemogenetically elicited SICs can elicit timing‐dependent plasticity.

Since astrocytic activation does not only cause glutamate release or alteration in glutamate uptake, the changes in EPSCs could have been the result of several various actions by astrocytic activation. To see whether glutamate release can elicit changes without other features of astrocytic activation, we released MNI‐caged glutamate by flash photolysis with variable timing compared to the evoked EPSCs. In 58% of all cases, we observed timing‐dependent changes of EPSCs (*n* = 17). If the peak of glutamate‐induced current preceded the EPSC (“negative shift”) or appeared at the same time with it, the amplitude was increased with 17%–116%. When the current followed the EPSC (“positive shift”), reduction of EPSC amplitude was observed (21%–26% decrease); thus, a similar weak STDP was elicited by excitatory currents elicited by glutamate (Figure 3f–h).

To investigate whether SICs alter synaptic strength as postsynaptic electrical signals, a previously recorded SIC was converted to a voltage command and applied with various timing. These artificial SICs evoked weak STDP‐like plasticity as it was seen with the previous manipulations (Figure 3i–k). EPSCs were changed by SIC commands in 44% of all cases (*n* = 36). The “negative” and “zero shifts” between the SIC command and the elicited EPSC caused 27%–177% increase in 35% of the cases and 26%–30% reduction in 10% of the cases. In case of positive shift, a 28%–45% decrease in EPSC amplitude was obtained without any increase (Table S3).

In conclusion, SICs have the capability to alter synaptic strength in a longer time scale acting as electrical signals on the postsynaptic neuron.

**FIGURE 4:**
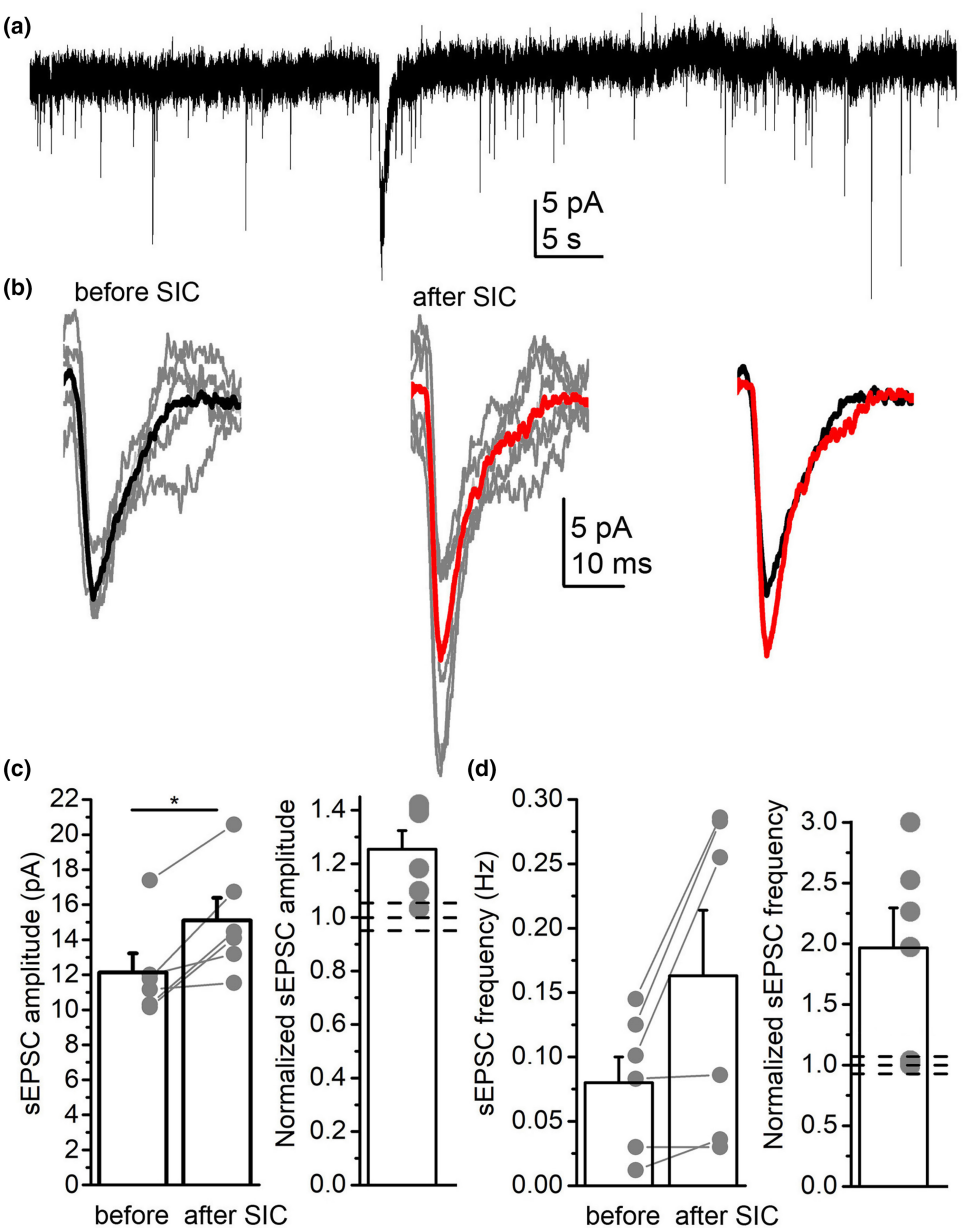
Human slow inward currents (SICs) also elicit synaptic plasticity. (a) A representative gap‐free current trace recorded from a human neocortical pyramidal neuron. (b) Average spontaneous EPSCs before and after the first detected SIC. Left: black: average EPSC of 32 individual EPSCs before the first SIC, gray: 5 representative individual EPSCs; middle: red: average EPSC of 51 individual EPSCs following the first SIC, gray: 5 representative individual EPSCs; right: superimposed images of average EPSCs before (black) and after the SIC (red). (c) Statistical analysis of spontaneous EPSC amplitudes before and after the first SIC (left). EPSC amplitudes after the SIC normalized to control values. (d) statistical analysis of sEPSC frequency with the same arrangement as in panel ‘c’. Hollow columns: average ± SEM, gray dots and lines: individual data; dashed lines: 100% ± 5% fluctuation limit; *p < 0.05.

As recordings above were performed in mice, we sought evidence whether similar mechanisms exist in humans. Recordings were performed in human samples with similar criteria as in the murine recordings with gap‐free protocol in magnesium‐free solution. We revealed a long lasting increase both in the amplitude and frequency of EPSCs after the first SIC. Those cases, where changes were below the 5% level calculated from spontaneous fluctuations were considered as cases without changes. The EPSC amplitudes showed 10%–42% increase in 83% of all cases (*n* = 6). In 66% of all cases, an increase of EPSC frequency was also seen. Although the frequencies before and after SICs did not differ significantly, 97%–200% increase was observed in four cases. In contrast to murine recordings, reduction failed to appear in any of the parameters (Table S4; Figure 4).

These results indicate that SICs as electrical signals from astrocytes to neurons can elicit a weaker form of STDP. This synaptic plasticity exists but might be different in humans as decrease was not observed.

### 3.4 Differences in murine and human aging of SICs and their actions on synaptic plasticity

In the next set of experiments, we investigated how aging alters SICs and their impact on synaptic plasticity in mice and humans.

**FIGURE 5:**
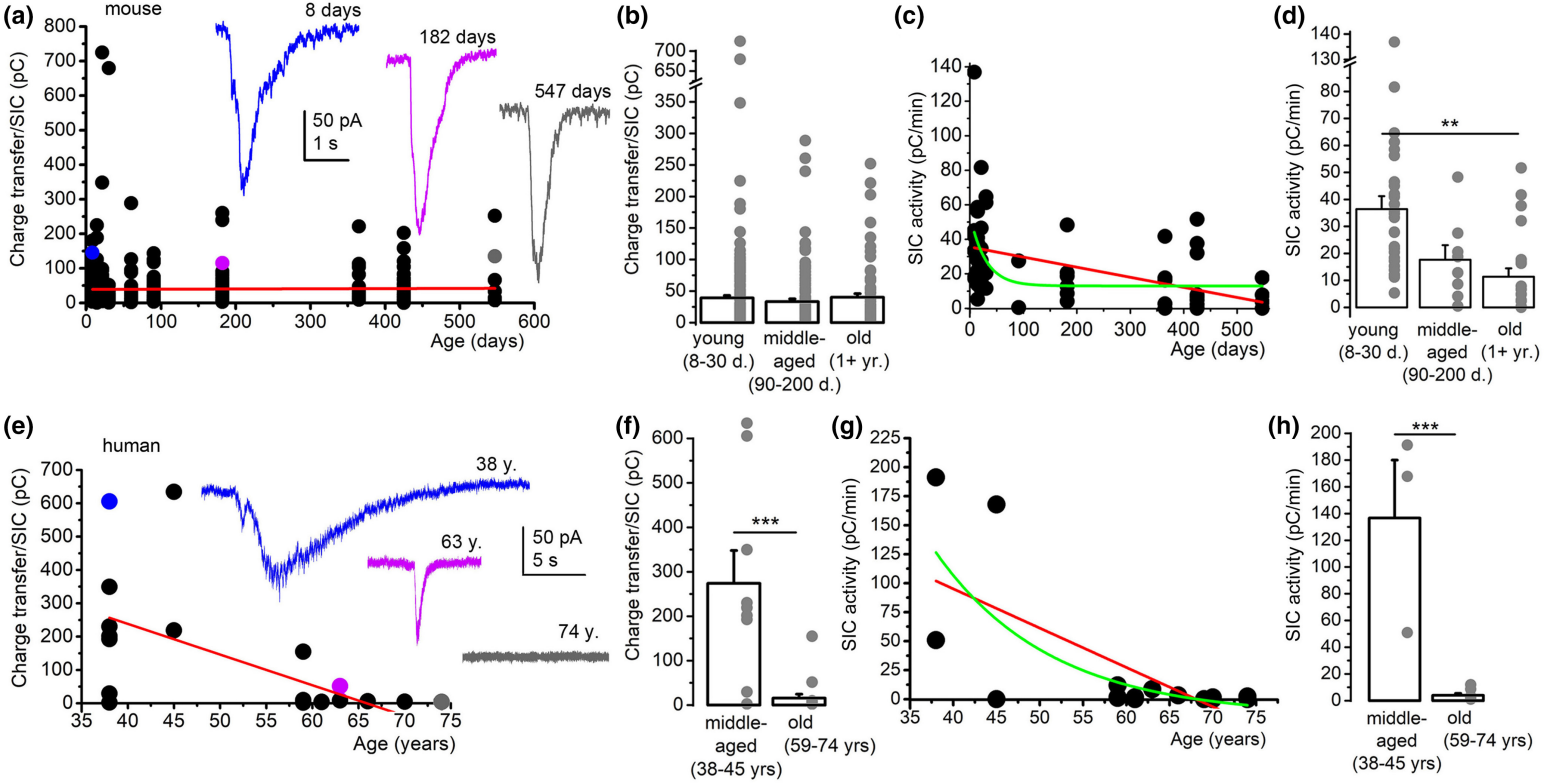
Age dependence of slow inward currents (SICs) in mice and humans. (a–d) Individual SICs do not decline with age, while the SIC activity declines in mice. (a) Charge transfer (the “area” of the individual SICs) plotted against the age of the mouse. Red line: linear fit of the dataset. Blue, purple and gray traces: representative SICs from mice with different ages. The corresponding dots in the graph are indicated with identical color. (b) Changes in charge transfer of individual SICs with age in three age ranges (hollow columns: average ± SEM, gray dots: individual data). (c) SIC activity (“area” of all SICs per minute) plotted against the age of the mouse. Black dots: individual data, red line: linear fit, green line: exponential fit. (d) Changes in SIC activity with age in 3 age ranges (hollow columns: average ± SEM, gray dots: individual data). (e–h) Both individual SICs and SIC activity declines with age in humans. (e) Charge transfer of the individual SICs plotted against the age of the patient (see panel ‘a’ for the arrangement). (f) Changes of SIC activity with age in three age ranges. Changes in individual SICs of different ranges of age. (g) SIC activity plotted against the age of the patient with the same arrangement as in panel ‘g, h’. **p < 0.01; ***p < 0.001.

Individual SICs were collected and analyzed from murine samples with different ages (from 9‐ to 547‐day‐old mice). The charge transfer of SICs did not change with age, but fluctuation of SIC amplitude, rise, and decay times was observed (Figure 5a,b; Figure S9A). SIC activity displayed a moderate age‐dependent decline due to the age‐dependent reduction of SIC frequency (Figure 5c,d; Figure S9A), but SIC activity was present at any age.

In contrast to mice, the charge transfer carried by human SICs showed a drastic decline with age and SICs completely disappeared above the age of 70 (Figure 5e,f). This decrease might be due to various reasons including the faster kinetics and lower frequency of senescent SICs (Figure S9B). In line with the decrease in charge transfer, SIC activity was also markedly declined with age and disappeared after 70 (Figure 5g,h). EPSC parameters did not decrease with age in any of the studied species (Figure S10).

**FIGURE 6:**
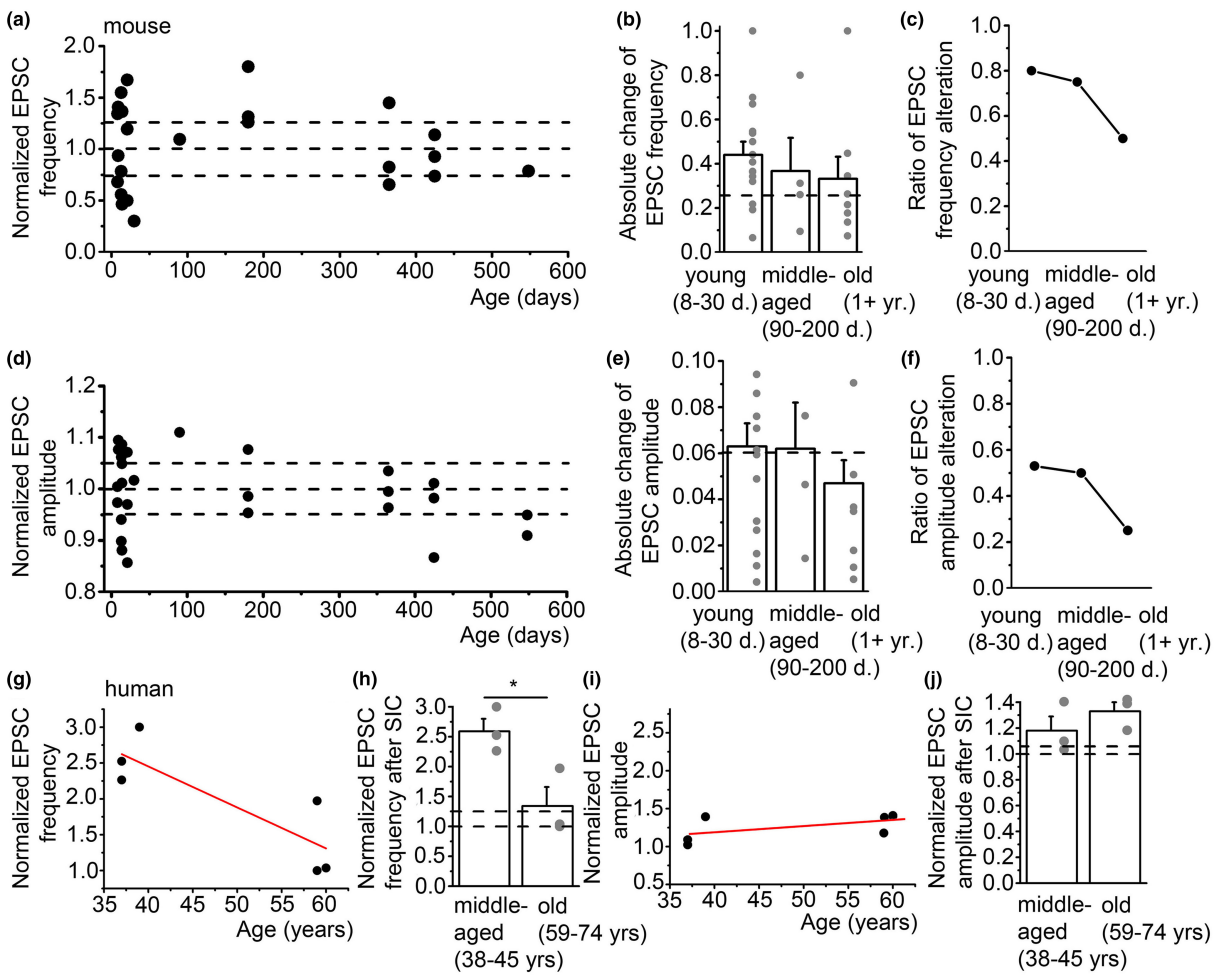
Age dependence of the capability of slow inward currents (SICs) to cause synaptic plasticity. (a–f) The proportion of EPSCs affected by SIC‐evoked synaptic plasticity declines with the age in mice. (a) EPSC frequency after the first SIC normalized to the EPSC frequency before the first SIC plotted against the age of the animal. Black dots: individual data, dashed lines: 100% ± 25% (range of fluctuation). (b) Absolute change of frequency (absolute difference of normalized EPSC frequency from 1) in different ranges of age (hollow columns: average ± SEM, gray dots: individual data). Dashed line: 25%, limit of fluctuations in control. (c) The ratio of data points in panel ‘b’ greater than 0.25 in different ages. (d, e) Changes in EPSC amplitude plotted with a similar arrangement as in ‘a–c’ except that the range of fluctuations is 6%. (g–j) The changes of EPSC frequency by SICs declines with age in humans. (g) Normalized EPSC frequency plotted against the age of the patient. Black dots: individual patient data, red lines: linear fit. (h) EPSC frequency after the first SIC normalized to the EPSC frequency before the first SIC plotted against the age of the patient (hollow columns: average ± SEM, gray dots: individual data). (i, j) Changes in EPSC frequency in different ages with the same arrangement as on panels ‘g, h’. Dashed lines 100% ± 25% (h) and 6% (j) as limits of spontaneous fluctuations. *p < 0.05.

Interestingly, the capability of SICs to elicit synaptic plasticity was differentially affected in mice and humans. In mice, there was a decline in the percentage of alterations in EPSC frequency and amplitude with age (Figure 6a–f). Averages of absolute changes in EPSC frequency and amplitude in different age groups showed a tendency of decline, but none of the datasets showed significant differences (Table S6; Figure 6a,b,d,e). However, the ratio of cases from all recordings where EPSC amplitude and frequency were altered by the first SIC showed a clear decline. This ratio for EPSC frequency was 0.8 for young, 0.75 for middle‐aged, and 0.5 for old mice (Figure 6c). For the EPSC amplitude, this ratio was 0.53 for young, 0.5 for middle‐aged, and 0.25 for old animals (Figure 6f).

In humans, such a decline was only observed in EPSC frequency, while no significant change was found in amplitudes. This observation might be biased by the fact that SICs fully disappeared above the age above 70; thus, their influence on EPSC parameters could not be investigated; therefore, we could involve less cases in this analysis (*n* = 6; Figure 6g–j). When EPSC frequencies after the first SIC were normalized to the ones prior to it, fitting the dataset and comparison of the normalized data from middle‐aged and old populations resulted a clear decrease (Figure 6g,h). Linear fit of the normalized EPSC amplitudes showed a tendency to increase with no significant differences between the two populations of age (Figure 6j; Table S7).

Changes in SIC activity and in synaptic responsiveness to SICs are partially the consequences of the change in GluN2B subunit expression. Compared to the GluN2B immunostaining colocalized with synaptophysin or GFAP in middle‐aged humans and mice, a decrease in the fluorescence intensity was seen in the old population (Figure S11).

In conclusion, individual SICs do not change in mice with age but a marked decrease of charge transfer is seen in humans. The overall SIC activity displays a mild reduction in mice due to the lower frequency. This reduction is much more pronounced in humans than in mice, as the SIC activity fully disappears in elderly in the former. The capability of SICs to elicit synaptic plasticity declines in both species but in a different way: In mice, the responsiveness to SICs is affected by aging, whereas the SIC activity itself is the subject of aging in humans.

## 4 DISCUSSION

In this project, we have confirmed that SICs exist in the human brain. We showed that these events differ from the ones recorded on mice. In contrast to mice, the charge transfer carried by human SICs is larger, which is mostly due to the kinetic differences observed in these events in the middle‐aged population. We have also demonstrated that SICs are capable of changing excitatory synaptic strength in a timing‐dependent way in largely half of the cases. This effect on synaptic plasticity is age‐dependent both in murine and in human samples as well. In mice, the size and kinetics of individual SICs do not decline with age but their frequency has a detectable decrease, their ability to change synaptic strength decreases with age. In humans, both the size and kinetics of SICs as well as their frequency decline with aging, leading to complete extinction of SIC activity above the age of 70.

### 4.1 Human SICs differ from murine SICs

When compared to murine SICs, we found that the charge transfer by human SICs was significantly greater but the amplitude tends to be smaller than in mice. Since we found a strong age dependence of SIC parameters in humans, we compared the middle‐aged mouse and human populations and showed slower kinetics, even though the amplitude of human SICs was significantly smaller. Kinetic differences are clear interspecies differences, whereas the reduced amplitude in humans might be partially the consequence of the different tissue handling.

It seems very likely that the astrocytic glutamate release properties and its diffusion pathways to the extrasynaptic NMDA receptors are responsible for the kinetic differences. Slower diffusion of glutamate might drastically alter the kinetic properties of extrasynaptic NMDA currents, even by modifying SICs to tonic currents in rodents (Carmignoto & Fellin, 2006; Fellin et al., 2004). Differences in the tortuosity of the glutamate diffusion pathways might be also different in mice and humans: It has been well demonstrated that human astrocytes have several morphological types with significantly more complex arborization of processes (Oberheim et al., 2009, 2012; Syková, 2004; Syková & Vargová, 2008).

### 4.2 SICs alone are capable of changing synaptic strength

Spike timing‐dependent plasticity (STDP) is a well characterized process of long‐term synaptic plasticity. The typical experimental arrangement for eliciting this phenomenon is the pairing of presynaptic stimulatory inputs with stimuli triggering postsynaptic action potentials, applied for several (usually 10–300) times in series (Inglebert & Debanne, 2021).

In the juvenile rodent hippocampus, when the postsynaptic action potential precedes presynaptic stimulation, LTD occurred, whereas LTP develops with the opposite arrangement (Mansvelder et al., 2019). In the adult human hippocampus, however, an intriguing time window shift of LTD‐LTP pattern was seen. If the time difference between the postsynaptic action potential and presynaptic stimulation was longer than −80 ms, LTD occurs, while in case of shorter time difference (including zero) LTP was detected (Testa‐Silva et al., 2010). In the human temporal cortex, compared to data obtained from juvenile rodent hippocampus, an opposite relationship between timing and shifts between presynaptic stimulation and postsynaptic action potentials was found. If postsynaptic action potentials occurred first, LTP developed, whereas in LTD was seen in opposite situations (Mansvelder et al., 2019; Verhoog et al., 2013).

The critical regulators of this shift between LTP and LTD are thought to be mechanisms based on the timing and duration of intracellular calcium signals. These alterations in intracellular calcium are due to the activation of voltage‐gated calcium channel, mGluRs and NMDA receptors by glutamate (Feldman, 2012; Inglebert & Debanne, 2021; Mizuno et al., 2001; Nevian & Sakmann, 2006).

There is growing body of evidence that astrocytes are capable of modulating long‐term synaptic plasticity (Sancho et al., 2021). A post‐before‐pre pairing protocol, which induced LTD in the hippocampus of the younger mice, induced LTP in mature ones. This switch and this form of LTP was mediated by NMDARs, but depended on NO release and mGluR activation. Astrocytic activation and consequential release of adenosine and glutamate were needed for the switch and LTP formation (Falcón‐Moya et al., 2020). The juvenile form of LTD is inhibited by astrocytic adenosine release after 1 month, whereas astrocytic glutamatergic signaling via NMDA‐ and metabotropic glutamate receptors changes suppression to LTP in the mature murine somatosensory cortex (Martínez‐Gallego et al., 2022).

Inhibiting glial functions alters long‐term synaptic plasticity. The mouse hippocampal LTP elicited by high frequency stimulation was reduced by the gliotoxin L‐α‐aminoadipate (L‐AA). The acute action of the gliotoxin was reverted by application of the gliotransmitter D‐serine (Pereira et al., 2021). Stimulation‐elicited LTP is decreased by decoupling of astrocytes in the mouse hippocampus (Hösli et al., 2022). Astrocyte‐regulated LTP of the mouse somatosensory cortex is affected in the mouse model of Alzheimer's disease and the consequential reduction of astrocytic Ca^2+^ signaling (Lia et al., 2023). The cognitive decline in aged mice is prevented in IP3KO mice with altered astrocytic calcium signaling (Guerra‐Gomes et al., 2018). In the mouse hippocampus, LTD was elicited by low frequency stimulation and LTP by high frequency stimulation. The gliotoxin L‐AA reduced the normal LTP amplitude but minimally affected the LTD amplitude. The amplitude of the LTP was reduced by amyloid β1‐42 exposure, and the LTD was converted to LTP. In case of Aβ1‐42 application, the LTP was not further reduced by the gliotoxin L‐AA but the Aβ‐induced LTP was reverted to LTD (Lopes et al., 2022).

Alterations of astrocytic glutamate uptake modify the NMDA‐evoked LTD in the mouse hippocampus (Naranjo et al., 2020). Astrocytes modulate the mouse hippocampal LTP by glutamate release and consequential activation of GluN2A‐containig synaptic NMDARs (Park et al., 2015). In a study performed on the mouse striatum, EAAT2 inhibition altered the STDP evoked by pairing protocol to an aberrant pattern due to the activation of extrasynaptic NMDARs (Valtcheva & Venance, 2016).

In a few recent studies, the capability of astrocytes to elicit long‐term synaptic plasticity alone was also demonstrated on mouse models. Astrocytic activity induced LTP in the mouse hippocampus which contributes to learning and memory recall (Adamsky et al., 2018). On the murine hippocampus, optogenetic astrocyte activation elicited LTD alone via ChR2 and it elicited LTP via Opto‐a1AR (Maltsev et al., 2023). In the mouse striatum, astrocytic activation and adenosin release elicits LTD by solely astrocytic activation. This fully astrocyte‐dependent LTD does not occlude the stimulation‐elicited neuronal LTD (Cavaccini et al., 2020).

In this project, we found that single SICs are able to elicit an anti‐Hebbian STDP in roughly half of the synapses. The reason for the plasticity does not follow Hebbian rules (i.e., if presynaptic stimulation precedes postsynaptic action potential, LTP is initiated) is very likely the protocol used for eliciting it. In typical STDP experiments, pairing protocols include short and repetitively (10–300 times) applied triggers to elicit 1–3 action potentials (Feldman, 2012; Inglebert & Debanne, 2021; Mizuno et al., 2001). SICs, considered as postsynaptic signals in our study, are prolonged resulting in a charge transfer of 1–634 pC (11 pC for the SIC as signal). Since postsynaptic calcium signals (via NMDA receptors and voltage‐gated calcium channels) are thought to be crucial for eliciting STDP (Inglebert & Debanne, 2021), we propose that a single SIC might lead to a prolonged elevation of intracellular calcium level at the time of presynaptic stimulation, which can trigger LTP when SICs precede the evoked EPSCs or when they appear simultaneously. If SICs are late compared to the evoked EPSCs, probably only the initial smaller and slower calcium increase may fit the time window for STDP. Those might act as “smaller” calcium signals leading to LTD (Inglebert & Debanne, 2021).

Our findings on human samples raise the possibility that single SICs can elicit synaptic plasticity in humans as well. However, by recording of spontaneous events one cannot exclude the possibility that we detected a network effect of a simultaneously occurring SICs on a neuronal population increasing the excitability of the whole local network instead of having only synaptic actions. Assuming that actions on synapses were seen in our experiments, it can be concluded that the impact of human SICs is different on synaptic plasticity, since while increases in frequency and amplitude were seen but suppression was not detected. This might be due to the known differences in murine and human STDP (Mansvelder et al., 2019), or could also be due to the greater and more prolonged charge transfer by human SICs compared to murine ones. The slower kinetics and greater charge transfer by human SICs might alter their role in regulation of synaptic plasticity since they cause slower and longer lasting increase in somatodendritic intracellular calcium concentration. Different calcium signals differentially affect spike timing‐dependent plasticity (Feldman, 2012; Inglebert & Debanne, 2021; Mizuno et al., 2001) which might explain the differences in our findings related to the aging of plasticity (see below).

### 4.3 SICs and SIC‐dependent changes in synaptic strength are affected by normal aging

Aging is associated with several morphological and functional alterations of astrocytes. There is no consensus how the number of GFAP‐positive astrocytes changes. In several studies, an increase was demonstrated in the rodent hippocampus (Bellaver et al., 2017; Cerbai et al., 2012; Goss et al., 1991; Kohama et al., 1995; Nichols et al., 1993) and neocortex, (David et al., 1997; Goss et al., 1991; Kohama et al., 1995; Nichols et al., 1993), as well as in the human hippocampus and striatum (Cerbai et al., 2012; David et al., 1997; Nichols et al., 1993). However, there were cases when the number of astrocytes decreased or did not change at all in the mouse hippocampus (Hayakawa et al., 2007) and in several brain areas of the macaque monkeys (Robillard et al., 2016). In contrast, other astrocytic markers like glutamine synthetase or S100B showed hardly any change in the mouse hippocampus (Rodríguez et al., 2014). Intriguingly, the morphology of individual astrocytes is affected by aging. The branch length and the complexity of astrocytic processes first increase to reach its maximum in adults, followed by a decrease in the hippocampi of aged primates and mice (Popov et al., 2021; Robillard et al., 2016). The volume sections of fine astrocytic processes shrink and the size of the astrocytic domains also decrease with age (Popov et al., 2021).

According to functional changes in astrocytic aging, no decrease was found in calcium oscillations, but the number of astrocytes with detectable oscillations was reduced (Gómez‐Gonzalo et al., 2017). The spread of calcium events had a shorter way (Popov et al., 2021). Aged astrocytes respond to stimuli since ionotropic and metabotropic glutamate receptors, P2X and P2Y receptors, acetylcholine, the protease‐associated receptor 1 (PAR‐1) agonist TFLLR, as well as noradrenaline and cannabinoid actions are activated in aged murine hippocampal and neocortical samples (Gómez‐Gonzalo et al., 2017; Lalo et al., 2011, 2018). On the contrary, a reduction in mGluR5‐mediated signaling was reported, probably as a consequence of the overall reduction of mGluR5 expression in the murine and human hippocampus and neocortex (Gómez‐Gonzalo et al., 2017; Sun et al., 2013).

Astrocytic capability to alter synaptic functions also changes with age. For aging of SICs, the following parameters should be considered: (a) calcium excitability and the number of astrocytes with calcium waves, (b) glutamate release and uptake by astrocytes, (c) diffusion of glutamate from the astrocytes to extrasynaptic NMDA receptors, (d) and the number of extrasynaptic NMDA receptors available for activation. (a) It was demonstrated that the astrocytic calcium excitability is not significantly altered by aging, but the number of reacting astrocytes decline (Gómez‐Gonzalo et al., 2017). This might be in line with our findings as only the SIC frequency is affected since there are less astrocytes to elicit them. (b) Age‐related decline of astrocyte‐specific EAAT‐1 and EAAT‐2 glutamate transporters was seen on the rodent hippocampus which increases ambient glutamate levels and thus weakens synaptic neurotransmission (Potier et al., 2010). Initially, there is an increase in glutamate transporter surface expression until it reaches its maximum in adults, followed by a slow decline with aging (Rǎdulescu et al., 2022). (c) According to the diffusion properties of glutamate, it is known that the increased tortuosity of the diffusion pathways can alter neuronal currents elicited by ambient glutamate (Syková, 2004; Syková & Vargová, 2008). As SICs become faster with age, it is in line with the observation that the complexity and volume of astrocytic processes decline with age, probably reducing the tortuosity of diffusion pathways leading to faster extrasynaptic neuronal events (Popov et al., 2021; Robillard et al., 2016). (d) The NMDA receptor surface expression and its functions also alter with age. NMDA receptor hypofunction, as well as a decline of calcium entry via NMDA receptors were reported in elderly (Foster, 2007; Nava‐Gómez et al., 2022). In rodents, an increase of extrasynaptic NMDA receptor expression was found (Rajani et al., 2021). However, GluN2B subunits are known to be abundant in neonates and a rapid decline of subunit expression together with an increase in GluN2A/GluN2B ratio was found in both rodents (Gramuntell et al., 2021; Liu et al., 2004) and humans (Law et al., 2003).

According to age‐related changes in murine SICs, we largely confirmed previous data claiming that individual SICs are not affected by aging. In line with data in literature, SICs persisted in the older ages of rodents, but the increase in SIC frequency was reduced with age (Gómez‐Gonzalo et al., 2017). In humans, however, parameters of individual SICs are also affected. The charge transfer and kinetic parameters have a strong decline in humans and SIC activity completely disappears after 70. This might be due to an alteration of astrocytic glutamate release or uptake, as well as extrasynaptic NMDA receptors (Gramuntell et al., 2021; Law et al., 2003; Liu et al., 2004; Potier et al., 2010; Rǎdulescu et al., 2022).

**FIGURE 7:**
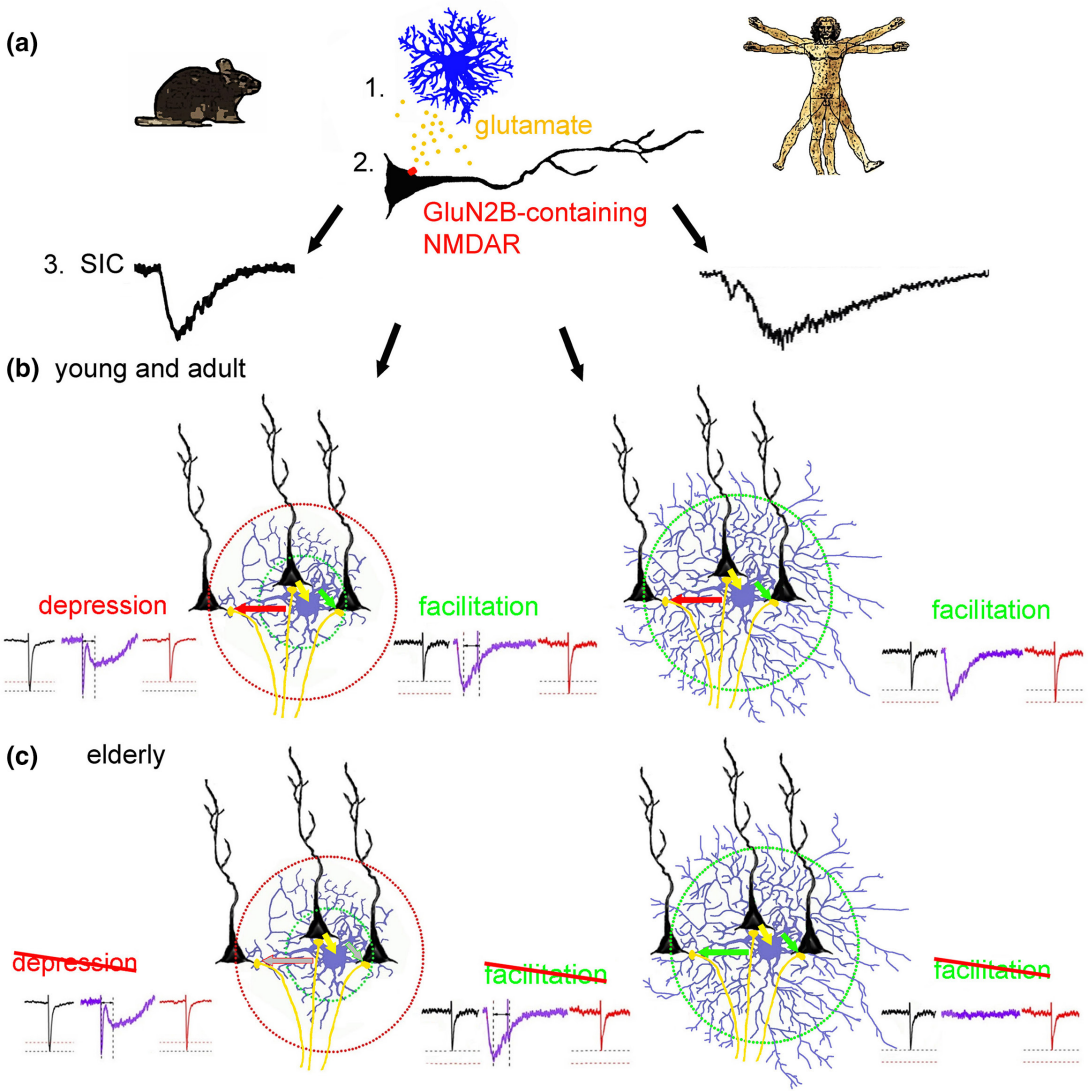
A summary of our findings. (a) Slow inward currents (SICs) are consequences of astrocytic glutamate release (1) and neuronal GluN2B‐containing NMDA receptor activation (2) both in mice (left) and human (right; see symbols). Murine and human SICs differ in amplitude and kinetic parameters (3). (b) In young and adult mice (left) timing‐dependent synaptic depression or potentiation occurs, possibly depending on the distance of synapses from the astrocytic glutamate release. In humans (right), only potentiation was seen, potentially due to the complexity of astrocytic morphology. (c) With aging, synaptic plasticity by SICs is reduced due to changes in synaptic responsiveness in mice (left), whereas SICs themselves are subject of aging in humans (right).

The actions of SICs on human and murine synaptic plasticity are differentially affected. In mice, the actions of SICs on synaptic plasticity are subjected to aging. This might be the consequence of the declined mGluR5 signaling or alterations of NMDA receptors by age. In humans, however, the SICs themselves are affected (due to glutamate release or uptake, or also by NMDA receptors). We demonstrated that there is a moderate but significant decline in GluN2B subunit density, which might contribute to the decline of SIC activity and synaptic responsiveness to SICs. However, due to the limited decrease, other factors should also contribute. These findings demonstrate the significant differences in murine and human brain aging (Figure 7).

### 4.4 SICs occurring with high frequency model pathological conditions

Under physiological conditions, SICs occur with low frequency (0.1 Hz or below). This frequency might question the significance of long‐term regulation of synaptic strength via SICs. However, as a rarely occurring homeostatic regulator, it might differentially affect synapses in different positions within the astrocytic domain: in the core of astrocytic glutamate release, it might have a stimulatory role; whereas laterally from the glutamate release, suppression might occur. If SICs are considered as physiologically occurring phenomena affecting synaptic plasticity, the decline in SIC activity might contribute to the cognitive decline in elderly.

Slow inward currents, as pathologically occurring events, might have a greater impact on synaptic plasticity. Under pathological conditions (e.g., epilepsy modeled by nominally magnesium‐free recording solution), SICs with relatively greater frequencies were recorded. A series of stochastically occurring large events with random timing to synaptic activity might cause an overall increase in synaptic activity or might randomly change synaptic strength. By doing it, it might significantly contribute to excitotoxicity or spreading depolarization. In addition, decline of SIC activity might be a benefit under pathological conditions, as this observed decline might be related to the decreased vulnerability of cortex to spreading depolarization with age (see Hertelendy et al., 2019).

## AUTHOR CONTRIBUTIONS

AC performed most murine electrophysiological experiments, AK performed most human electrophysiological recordings. BM operated and handled mice for DREADD experiments and contributed to electrophysiological recordings. KP did the morphological analysis of the recorded neurons. KK performed electrophysiological experiments and analyzed the results. AK provided the human samples. PS analyzed the results and wrote the manuscript. PPN wrote the manuscript. BP initiated the project, analyzed the results, and wrote the manuscript.

## FUNDING INFORMATION

This work was funded by the National Research Development and Innovation Office (NKFIH‐K138090 to PN) and by the project no. TKP2020‐NKA‐04 which has been implemented with the support provided from the National Research, Development and Innovation Fund of Hungary, financed under the 2020‐4.1.1‐TKP2020 funding scheme. The project was also supported by the Hungarian National Brain Research Program (KTIA_13_NAP‐A‐I/10, BP). AK was supported by the 2017‐1.2.1‐NKP‐2017‐00002 National Brain Research Program NAP 2.0. PS was supported by the Hungarian Brain Research Program (KTIA_NAP_13‐2‐2014‐0005; 2017‐1.2.1‐NKP‐2017‐00002). BM was supported by the Stipendium Hungaricum PhD program.

## CONFLICT OF INTEREST STATEMENT

The authors have declared that no conflict of interest exists.

## Supporting information


Appendix S1.

## Data Availability

All data are available in the main text and the supplementary material in a graphical form. Numerical data will be made available on reasonable request.
