# Supplementary material for: Astrocyte‐ and NMDA receptor‐dependent slow inward currents differently contribute to synaptic plasticity in an age‐dependent manner in mouse and human neocortex
Source: Aging Cell. 2023 Jul 25;22(9):e13939. doi: 10.1111/acel.13939 (PMC10497838; doi:10.1111/acel.13939)
Supplement: Supplementary file 1 — Appendix S1. [file ACEL-22-e13939-s001.docx]

**Supplementary material**


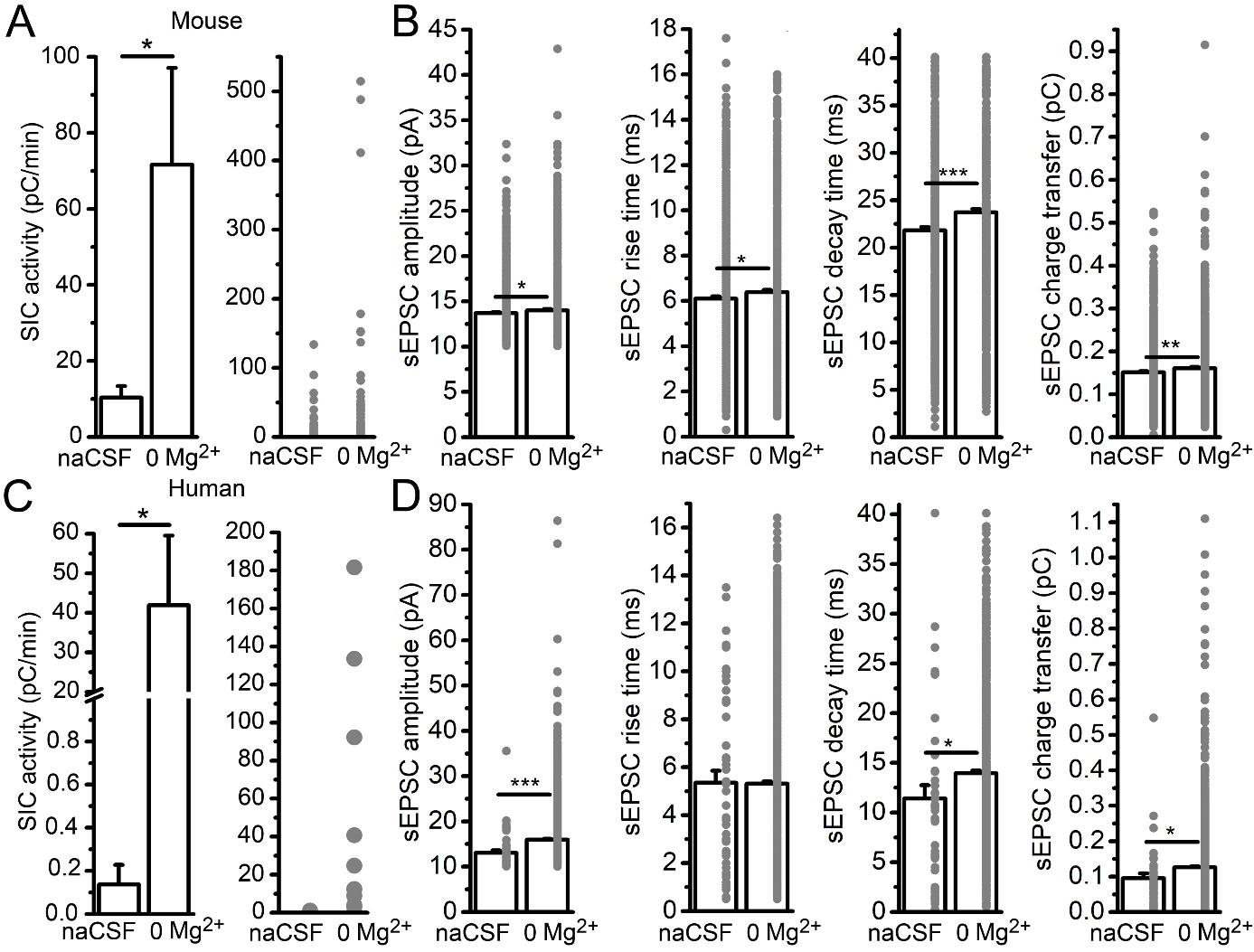


**Suppl. Fig.1. The magnesium-free recording solution significantly increases SIC activity and EPSC parameters both on mouse and human pyramidal neurons.** A. Statistical comparison of mouse SIC activity (i.e. charge transfer by SICs in a minute) in the presence of 1 mM Mg^2+^ (naCSF) and in nominally magnesium-free aCSF (left, hollow columns: average ± SEM; right, grey dots: individual data). In naCSF: 10.36 ± 3.05 pC/min; in magnesium-free solution: 71.59 ± 25.46 pC/min; p = 0.015; n = 140 neurons. B. Statistical comparison of sEPSC parameters under the same conditions as on panel A (hollow columns: average ± SEM, grey dots: individual data). Note that the amplitude, rise and decay time, as well as the charge transfer (‘area’) of mouse EPSCs were significantly increased. sEPSC amplitude: 13.7 ± 0.1 pA in naCSF; 14 ± 0.13 pA in magnesium-free solution; p = 0.039. Rise time: 6.1 ± 0.1 ms in naCSF; 6.38 ± 0.13 ms in magnesium-free solution; p = 0.029. Decay time: 21.83 ± 4.35 ms in naCSF; 23.72 ± 0.36 ms in magnesium-free solution; p < 0.0001. Charge transfer: 0.152 ± 0.0024 pC in naCSF; 0.161 ± 0.0027 pC in magnesium-free solution; p = 0.0049; n = 2333 events. C-D. Changes of SIC activity and EPSC parameters of human samples in magnesium-free recording solution, respectively. The arrangement of the panels is the same as on A-B. Note that the amplitude increase is greater but the prolongation of rise time is missing on human neurons. SIC activity: 136.9 ± 90.8 pC/min in naCSF; 41.93 ± 17.58 pC/min in magnesium-free solution; p = 0.01; n = 25 neurons. sEPSC amplitude: 13.06 ± 0.54 pA in naCSF; 15.97 ± 0.19 pA in magnesium-free solution; p = 0.0009. Rise time: 5.35 ± 0.5 ms in naCSF; 5.31 ± 0.1 ms in magnesium-free solution. Decay time: 11.41 ± 1.33 ms in naCSF; 13.95 ± 0.27 ms in magnesium-free solution; p = 0.03. Charge transfer: 0.096 ± 0.014 pC in naCSF; 0.126 ± 0.0028 pC in magnesium-free solution; p = 0.017; n = 1426 events. (*: p<0.05; **: p<0.01; ***: p<0.001) ***For Fig.1.***

***
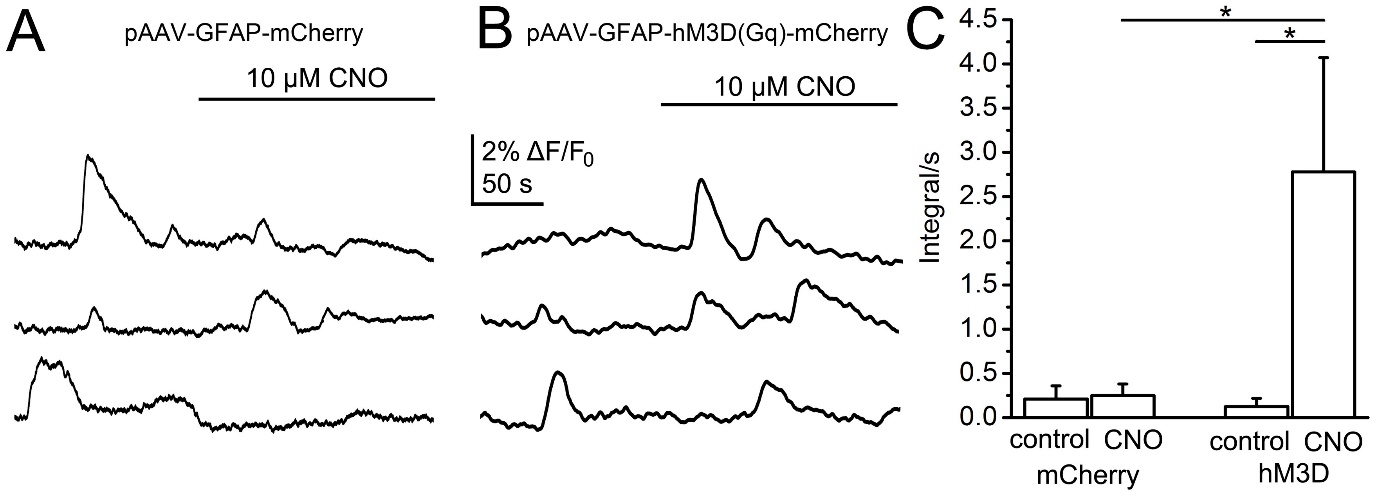
***

**Suppl. Fig. 2. The chemogenetic activation of astrocytes increases Ca^2+^-wave activity of astrocytes.**  A. Representative calcium wave traces from samples expressing mCherry tag under GFAP promoter from 3 individual regions of interest. B. Representative calcium wave traces from samples expressing hM3D(Gq) chemogenetic actuator under GFAP promoter from 3 individual recordings. C. Statistical summary of changes in calcium wave areas from different samples under different conditions. In mCherry expressing samples under control conditions: 0.21 ± 0.15 % ΔF/F_0_/s, in CNO: 0.25 ± 0.13 % ΔF/F_0_/s; in hM3D expressing samples under control conditions: 0.125 ± 0.094 % ΔF/F_0_/s, in CNO: 2.78 ± 1.29 % ΔF/F_0_/s; p (between different samples treated with CNO) = 0.044; p (between control and CNO treated hM3D expressing samples) = 0.032; n = 8-8 in both groups (hollow columns: average ± SEM; *: p<0.05) ***For Fig.1.***

**
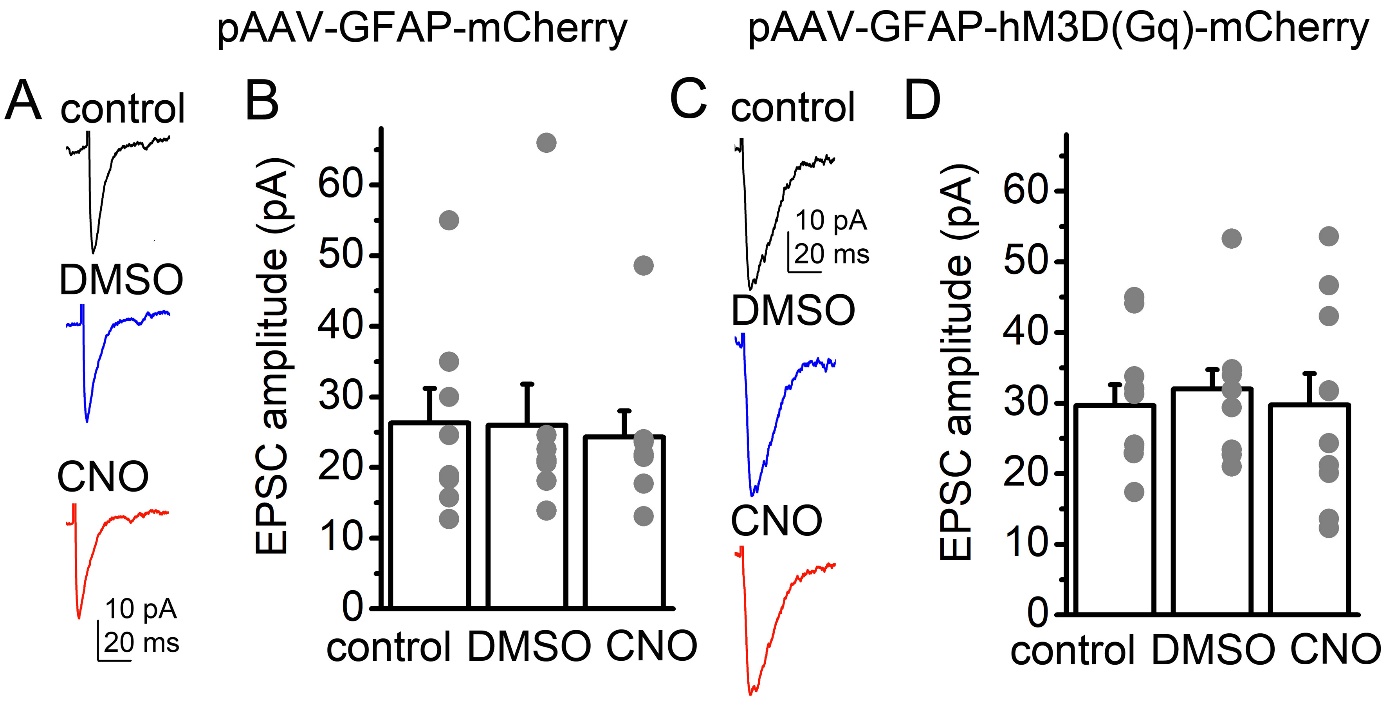
**

**Suppl. Fig.3. Neither the clozapine-N-oxide itself nor the chemogenetic activation of astrocytes changes evoked EPSC amplitude.** A. Average of 10 evoked EPSCs recorded under control conditions (black), with 0.1% DMSO (blue) and 10 µM CNO from samples of mice expressing mCherry tag under GFAP promoter. B. Statistical comparison of the EPSC amplitudes under the conditions presented on panel A (hollow columns: average ± SEM, grey dots: individual data). naCSF control: 26.3 ± 4.9 pA; DMSO control: 26 ± 5.82 pA; CNO: 24.3 ± 3.72 pA. C. Average of 10 evoked EPSCs recorded under control conditions (black), with 0.1% DMSO (blue) and 10 µM CNO from samples of mice expressing hM3D(Gq) under GFAP promoter. D. Statistical analysis of EPSC amplitudes under the conditions presented on panel C with similar arrangement as on panel B. naCSF control: 29.65 ± 2.96 pA; DMSO control: 32 ± 2.93 pA; CNO: 29.76 ± 4.45 pA. The number of astrocytes within 70 µm (this is the distance from which the astocytic processes potentially reach the neuronal soma; Kovács and Pál, 2017) from the soma was 3.67 ± 0.76 in case of hM3D-expressing samples and 4.66 ± 0.88 in exclusively mCherry-expressing control. No significant difference was seen between athe astrocytic numbers between the two populations (p = 0.22) ***For Fig.1.***


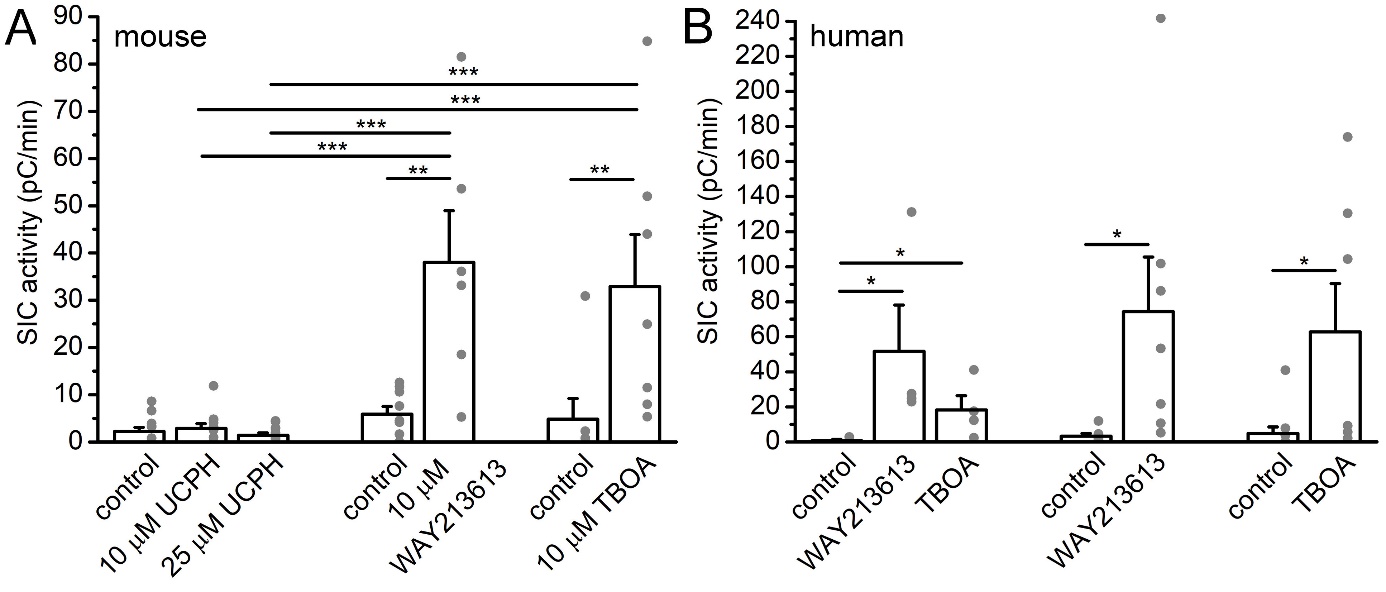


**Suppl. Fig. 4. The EAAT2 specific glutamate transport inhibitor WAY213613 has the same action on SIC activity as the non-specific EAAT inhibitor DL-TBOA. A.** Statistical comparison of the EAAT1 specific inhibitor UCPH101 (n = 12) on SIC activity in different concentrations and with actions of WAY213613 (n = 11) and DL-TBOA (n = 7). No actions of UCPH101 were seen on SIC activity (hollow columns: average ± SEM, grey dots: individual data) **B**. Statistical comparison of actions on SIC activity by various EAAT inhibitors applied one after the other (first group; WAY213613: EAAT2-specific inhibitor; DL-TBOA: non-specific EAAT inhibitor P = 0.05 for WAY213613 and 0.038 for DL-TBOA; n = 4) or alone on human samples (p = 0.02 for WAY213613 and p = 0.025 for DL-TBOA). (*: p<0.05; **: p<0.01; ***: p<0.001) ***For Fig. 1 and 2.***


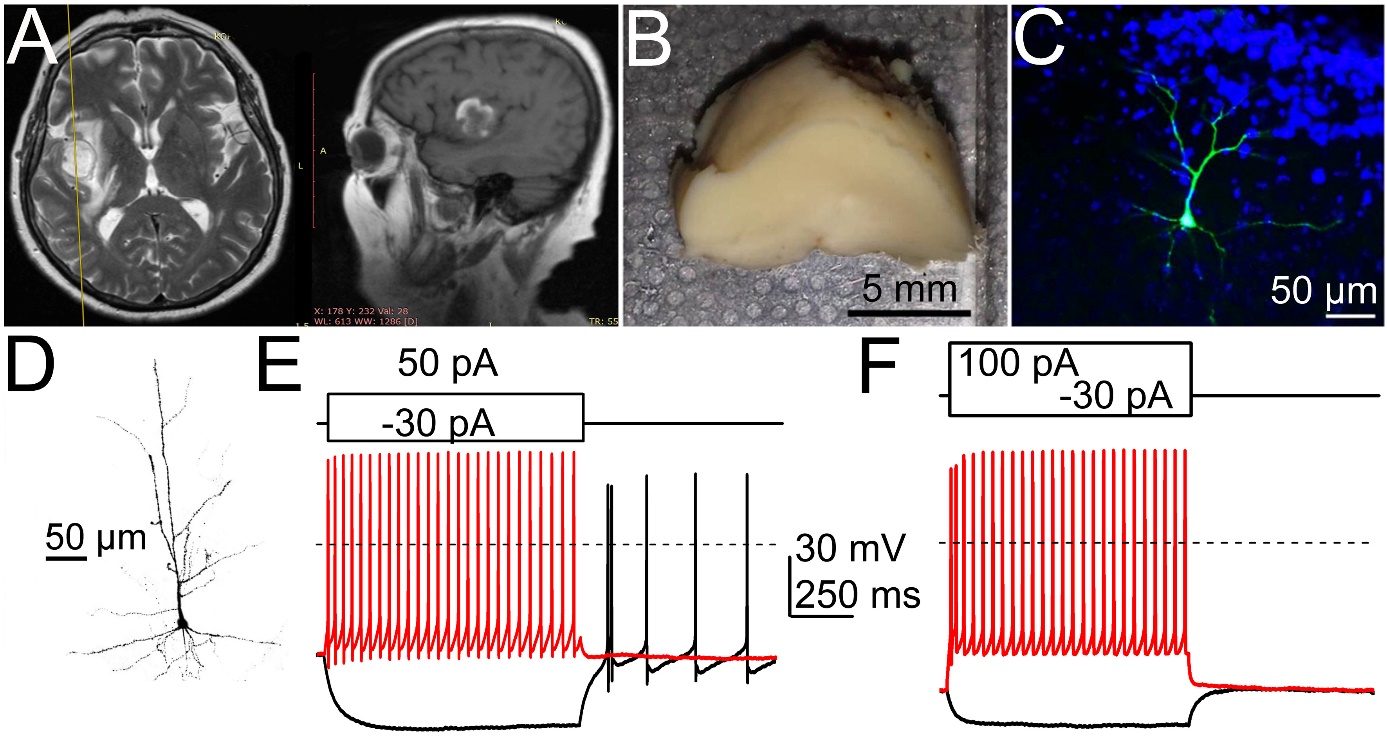


**Suppl. Fig.5. Collection of human samples and recordings from neocortical pyramidal neurons.** A. Location of the tumor on computer tomographic images. The right image was taken in the plane indicated with the yellow line on the left. Samples were taken from the area removed for the approach of the tumor. B. A human neocortical sample transferred from the operation theatre. C. Z-stack confocal image of a pyramidal neuron filled with biocytin (green; blue: DAPI staining). D. An image of a pyramidal neuron labelled with biocytin after merging 12 z-stack images. E-F. Current clamp recordings from pyramidal cells (black: voltage traces elicited with -30 pA current injection; red: voltage traces recorded with 50 or 100 pA depolarizing current injections). ***For Fig. 2.***

**
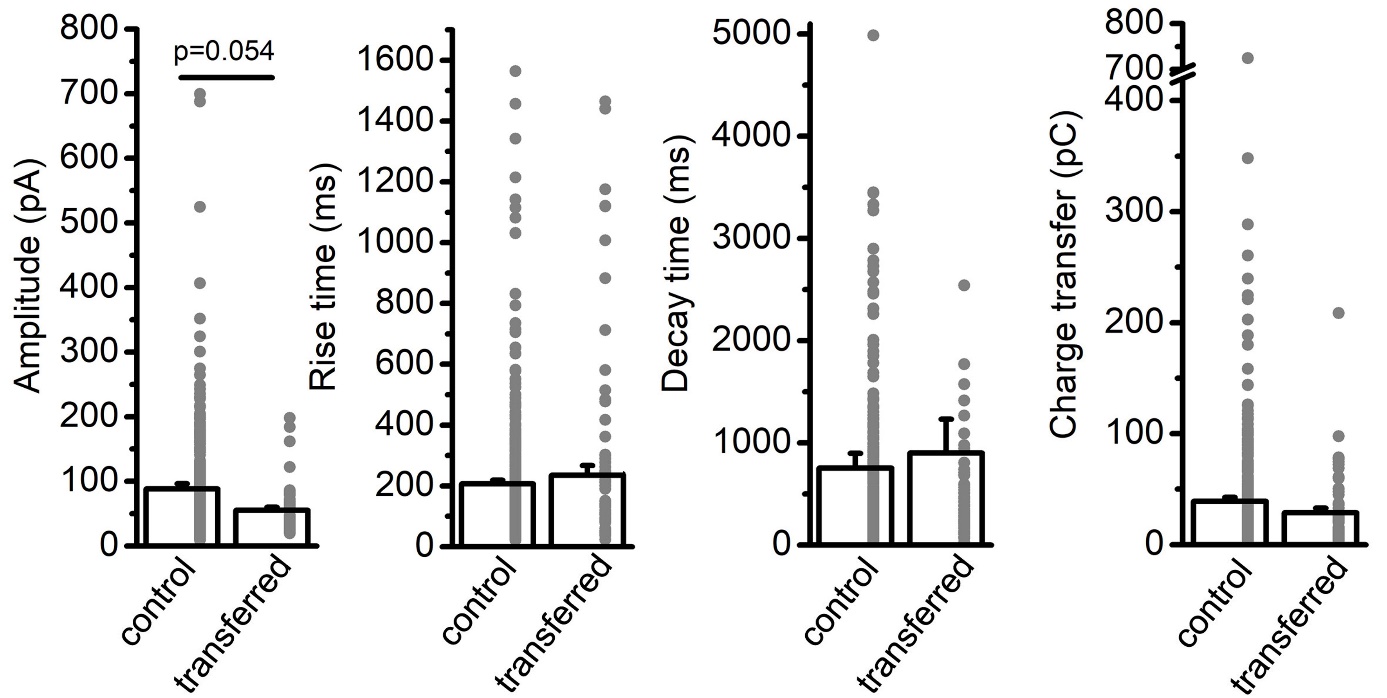
**

**Suppl. Fig. 6. Comparison of SIC parameters from mouse samples prepared under standard laboratory conditions (control) and under conditions modelling the transport of human specimen from the operating theatre to the laboratory (‘transferred’).** Recordings were done in nominally magnesium-free aCSF. Note that there is a tendency for amplitude reduction with near-significant difference between the two datasets, thus there might be a mild underestimation of human SIC amplitudes (hollow columns: average ± SEM, grey dots and lines: individual data). SIC amplitudes: standard incubation (control): 88.16 ± 8.14 pA, transferred samples: 55.02 ± 4.74 pA, p = 0.054. Rise time: control: 206.28 ± 12.28 ms, transferred: 234.8 ± 31.8 ms. Decay tau: control: 754 ± 143.85 ms, transferred: 901 ± 331 ms, charge transfer: control: 39.16 ± 3.45 pC, transferred: 28.64 ± 4.37 pC. ***For Fig. 2.***

**
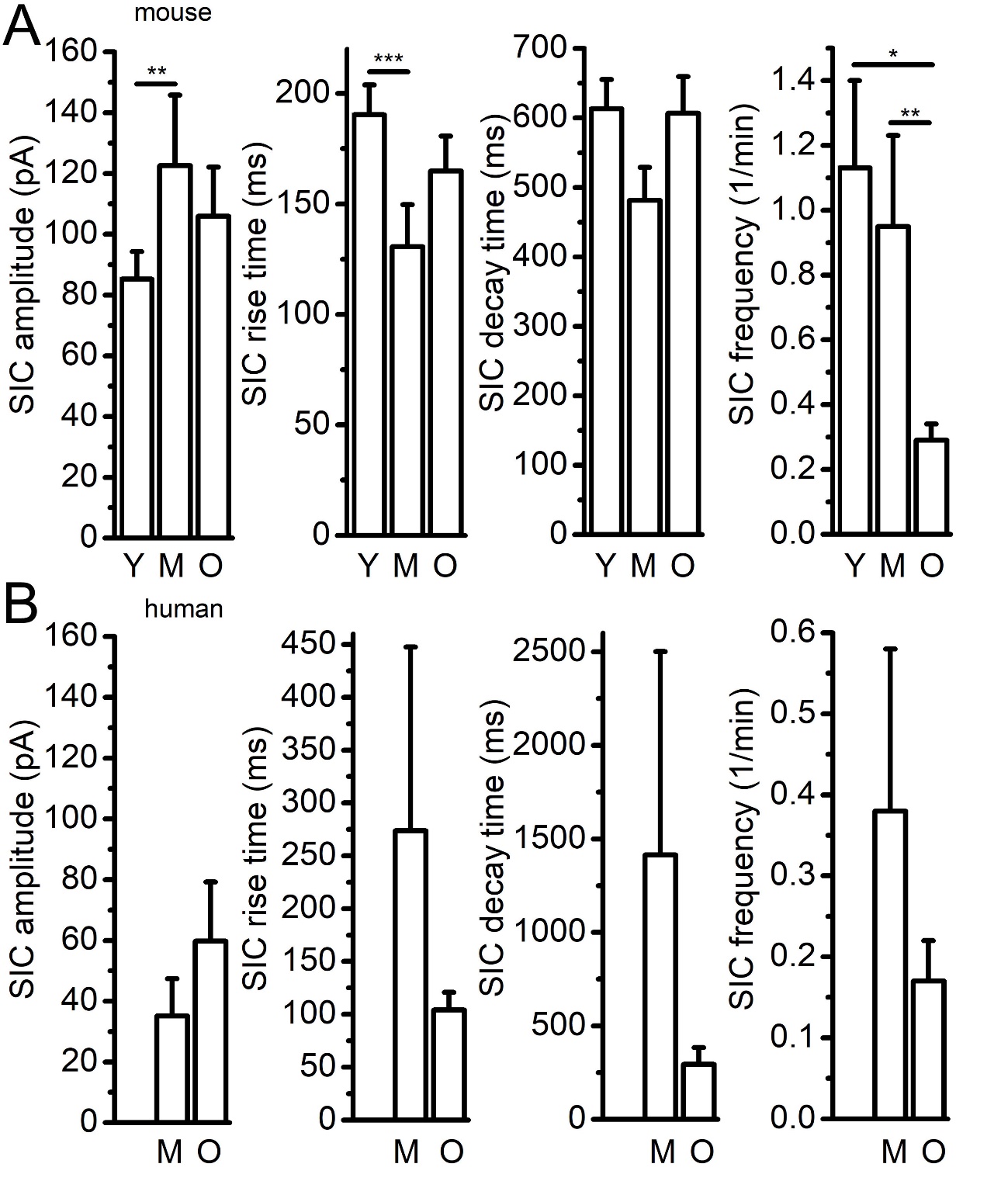
Suppl. Fig. 7. Human SICs have a significantly smaller amplitude but greater decay time and area in the middle-aged populations.** (hollow columns: average ± SEM; **: p<0.01; ***: p<0.001). SIC amplitude: human: 31.2 ± 11.5 pA, murine: 107.8 ± 8.25 pA, p = 0.0065; rise time: human: 239.6 ± 154 ms, murine: 153.8 ± 20.4 ms; decay time: human: 1.26 ± 0.97 s, murine: 0.47 ± 0.053 s, p = 0.0042; charge transfer: human: 246.51 ± 71.75 pC, murine: 31.03 ± 4.5 pC, p < 0.0001; n = 9 human and 95 murine SICs. Middle-aged mice: 90-200-day-old ; human: 38-45 yrs. ***For Fig. 2.***

**
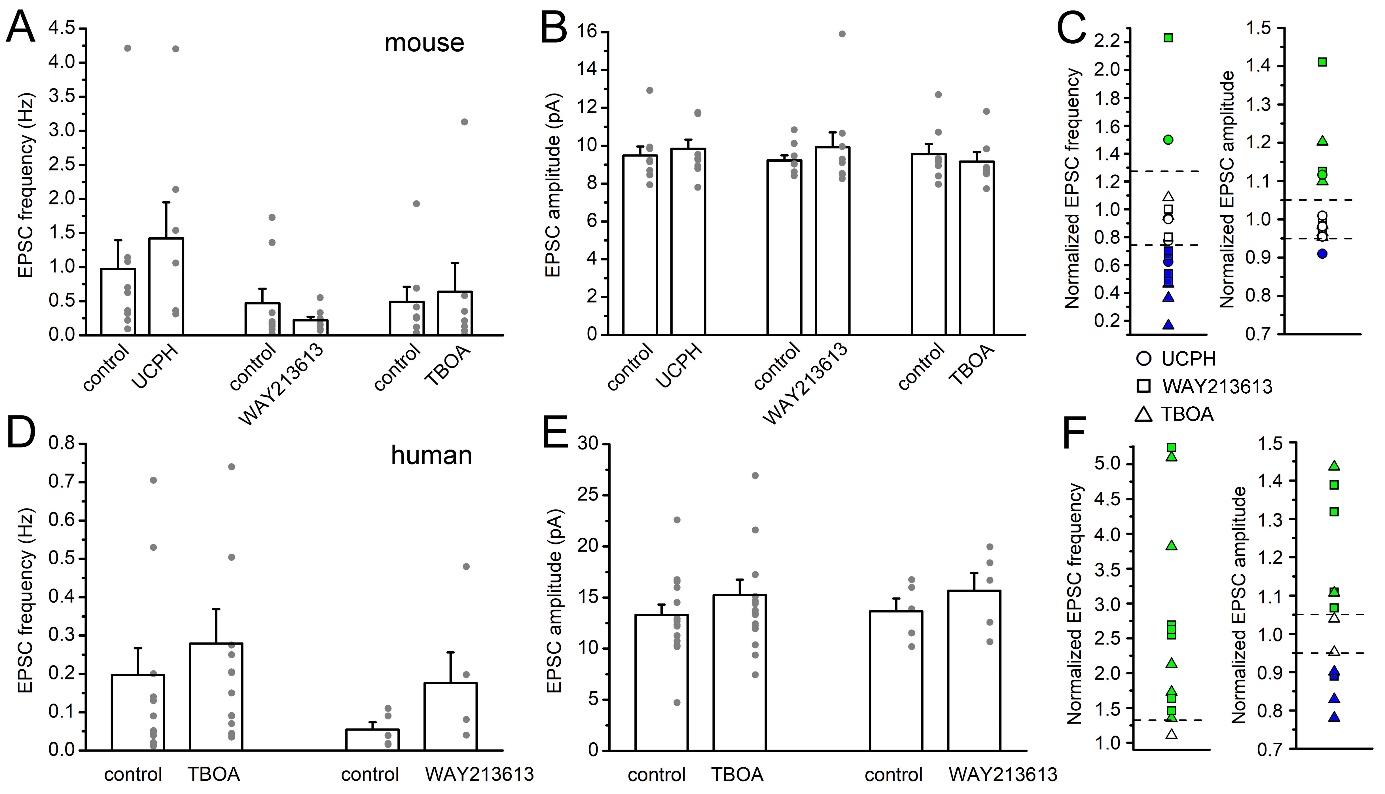
**

**Suppl. Fig. 8. Increased ambient glutamate concentration did not fundamentally alter the actions of SICs on synaptic plasticity. A-B.** The EPSC frequency (A) and amplitude (B) were not changed by EAAT inhibitors on murine samples. **C.** Increased ambient glutamate concentrations did not alter actions of SICs on synaptic plasticity on mice (circles: UCPH101 action; square: WAY213613 action; triangle: DL-TBOA action). In juvenile mice with spontaneously occurring SICs, 6.3 ± 1,1% absolute change of frequency and 44.5 ± 6% absolute change of amplitude were recorded. When DL-TBOA or WAY213613 application elicited the first SICs, these changes were 8.3 ± 3.4% and 43.2 ± 10.2%. No significant differences were found between the absolute changes of the frequency and amplitude (p = 0.275 and 0.457, respectively). In humans, spontaneously occurring SICs resulted 25 ± 7% change in amplitude and 96.6 ± 33% in frequency. SICs elicited by inhibition of glutamate uptake resulted 19 ± 4% change in amplitude and 181 ± 11% in frequency. No significant differences were found between the two experimental situations (p = 0.21 for amplitude and 0.11 for frequency). **D-E.** sEPSC frequency (D) and amplitude (E) only showed a tendency of increase by inhibition of astrocytic glutamate uptake in humans. **F.** EPSC frequency was increased by SICs elicited by EAAT inhibition; whereas the EPSC amplitude decreased in a few cases. ***For Fig. 2 and 3.***

**
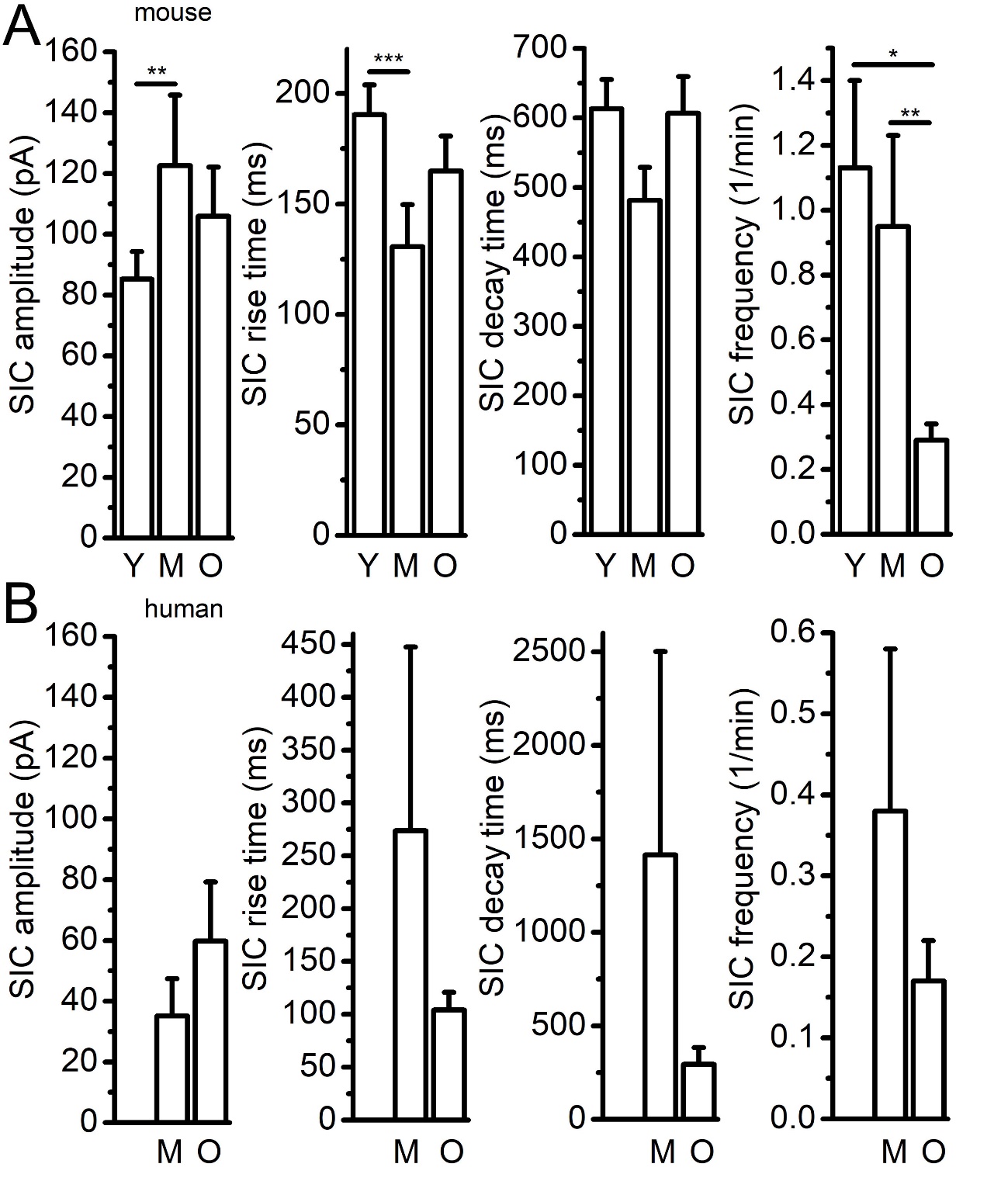
Suppl. Fig. 9. Changes of different SIC parameters with age in mouse and human.** A. Changes of SIC amplitude, rise and decay time and SIC frequency with age (Y: young, 8-30-day-old; M: middle-aged, 90-200-day-old; O: old, above 1 year). Hollow columns: average ± SEM. SIC amplitudes: young: 85.25 ± 9.05 pA, middle-aged: 122.63 ± 23.2 pA; old: 105.9 ± 16.26 pA, p (young vs. old) = 0.0027; rise time: young: 190.32 ± 13.58 ms, middle-aged: 130.66 ± 19.03 ms, old: 164.9 ± 15.8 ms, p (young vs. middle-aged) = 0.0008; decay time: young: 613.45 ± 42.06 ms, middle-aged: 481.5 ± 47.5 ms, old: 606.9 ± 52.76 ms; frequency: young: 1.13 ± 0.27/min, middle-aged: 0.95 ± 0.28/min, old: 0.29 ± 0.05/min, p (young vs. old) = 0.021, p (middle-aged vs. old) = 0.002. B. Changes of the SIC parameters with age in humans (M: middle-aged, 38-45-year-old; O: old, 59-74-year-old). SIC amplitude: middle-aged: 35.15 ± 12.27 pA, old: 59.8 ± 19.41 pA; rise time: middle-aged: 273.8 ± 104.1 ms, old: 173.8 ± 16.62 ms; decay tau: 1413.9 ± 1087 ms, old: 294.3 ± 89.11 ms; frequency: middle-aged: 0.38 ± 0.2/min, old: 0.17 ± 0.05/min. (*: p<0.05; **: p<0.01; ***: p<0.001) ***For Fig. 5.***

**
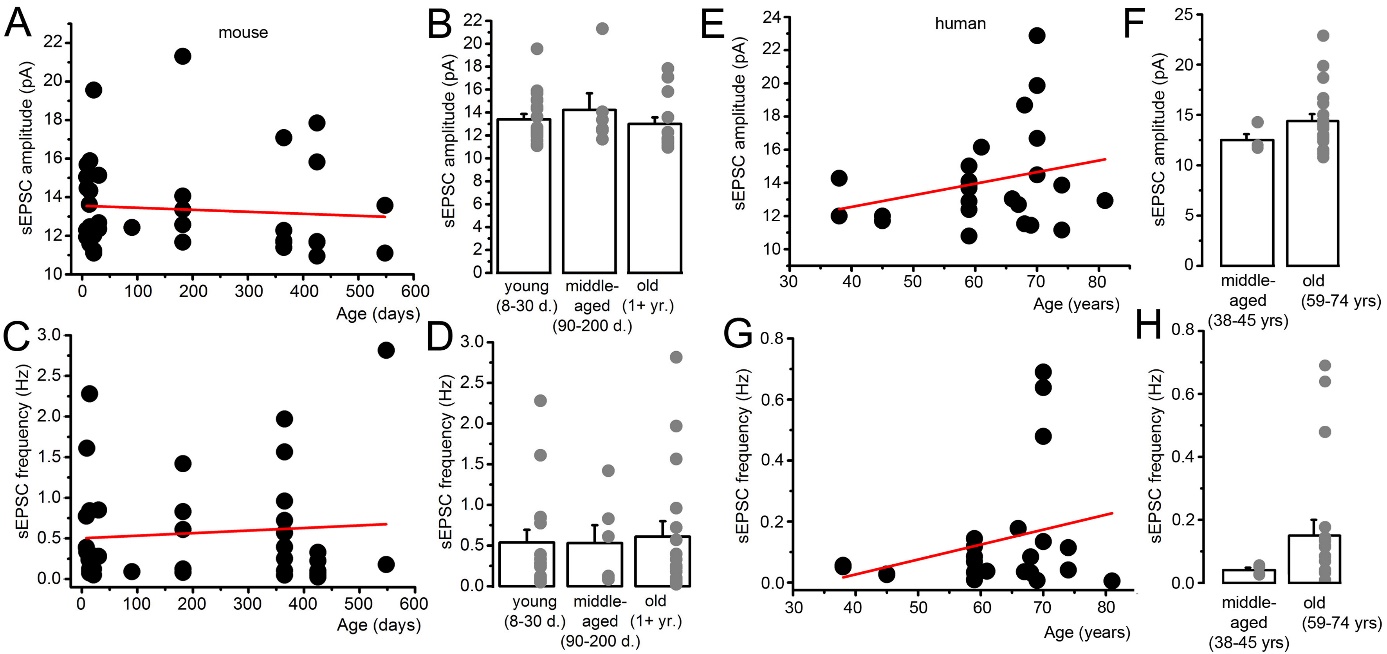
**

**Suppl. Fig. 10. There are no changes in sEPSC amplitudes with age.** A-D. sEPSC amplitude and frequency does not change with age in mice. A. sEPSC amplitude plotted against the age of mice (black dots: individual average EPSC amplitudes, red line: linear fit of the dataset; r^2^ = -0.0176; slope = -0.001 pA/day). B. Changes of EPSC amplitude with age in 3 age ranges (young: 13.4 ± 0.47 pA, middle-aged: 14.23 ± 1.45 pA, old: 13 ± 0.57 pA, n = 63; hollow columns: average ± SEM, grey dots: individual data). C-D. Changes of sEPSC frequency with age. The arrangement of the panels is the same as A-B. (C. r^2^ = 0.019, slope = 0.000315 Hz/day. D. young: 0.538 ± 0.155 Hz, middle-aged: 0.53 ± 0.22 Hz, old: 0.61 ± 0.19 Hz)

E-H. Similar to mice, human sEPSCs are also unchanged with age. For panel arrangement, see A-D. (E. r^2^ = 0.027, slope = 0.07 pA/year. F. middle-aged: 12.5 ± 0.6 pA, old: 14.4 ± 0.69 pA, n = 24. G. r^2^ = 0.039, slope = 0.00489 Hz/years. H. middle-aged: 0.04 ± 0.077 Hz, old: 0.15 ± 0.005 Hz.) ***For Fig. 5.***


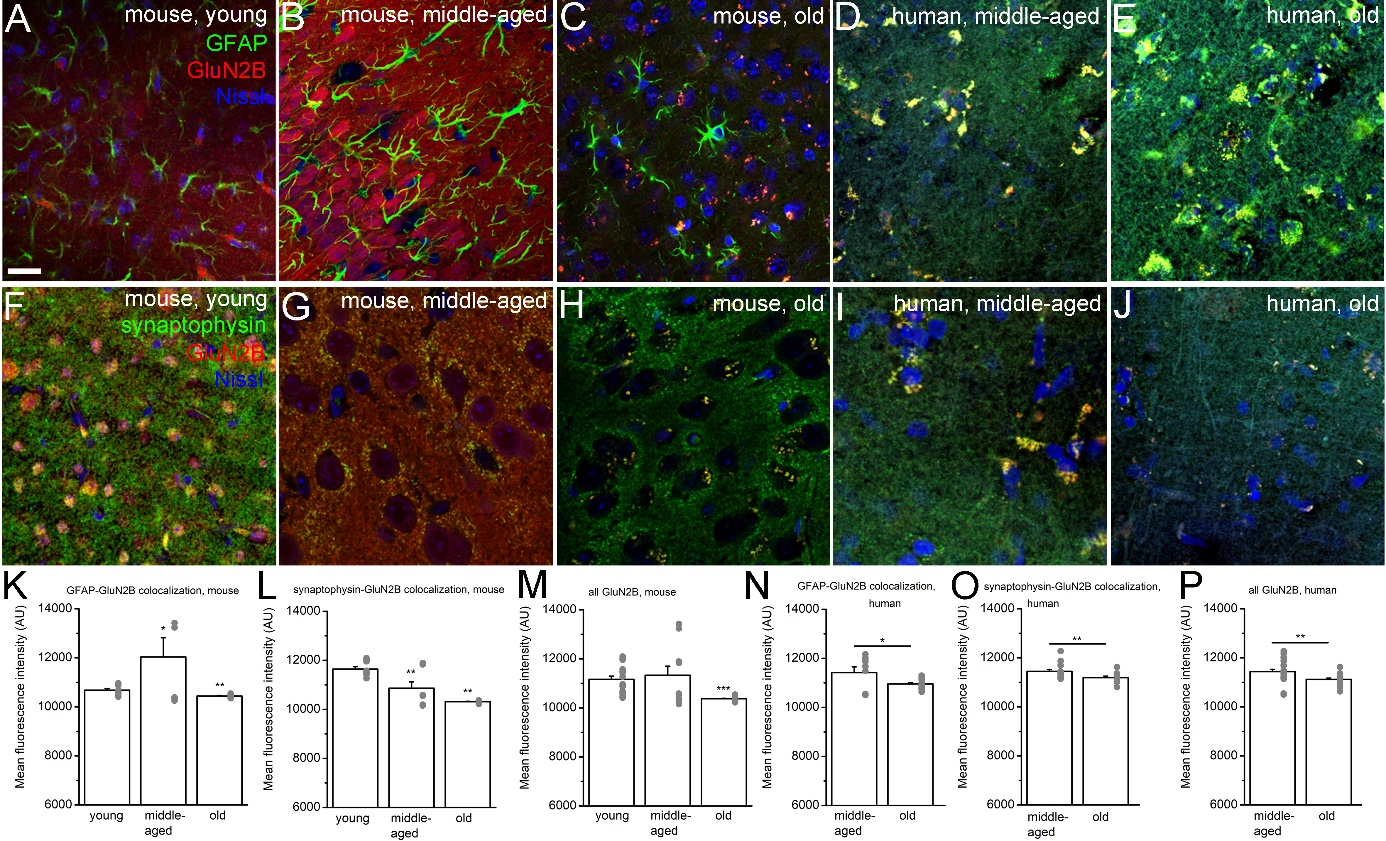


**Suppl. Fig. 11.**  **Changes of GluN2B subunit in synapses and next to astrocytes with age.** A-C. Merged immunohistochemical images with GFAP staining of the neocortex from young (14 days old), middle-aged (90 days old) and old (585 days old) mice, respectively (GFAP: green, GluN2B: red, Nissl and DAPI: blue). D-E. Merged immunohistochemical images with GFAP staining of the neocortex from middle-aged (38 years old) and old (69 years old) humans with the same color arrangement as on panels A-C. Note that all human samples used for this experiment were fixed under suboptimal conditions, as samples were from slice electrophysiology experiments. F-H. Merged immunohistochemical images with synaptophysin staining of the neocortex from young, middle-aged and old mice, respectively (synaptophysin: green, GluN2B: red, Nissl and DAPI: blue). I-J. Merged immunohistochemical images with GFAP staining of the neocortex from middle-aged (38 years old) and old (68 years old) humans with the same color arrangement as on panels F-H. K-M. Statistical analysis of red fluorescence intensities colocalized with green fluorescence on murine samples (GFAP on panel K, synaptophysin on panel L and both on panel M; columns: average ± SEM, grey dots: individual samples). N-P. Statistical analysis of red fluorescence intensities colocalized with green fluorescence on murine samples (GFAP on panel N, synaptophysin on panel O and both on panel P; columns: average ± SEM, grey dots: individual samples). Scale bar for all images: 20 µm. ***For Fig. 5 and 6.***

| **Mouse** | | | | | |
| --- | --- | --- | --- | --- | --- |
| ***Parameter*** | **SIC** | | **EPSC** | | |
|  | Average ± SEM | range | Average ± SEM | range | |
| ***Amplitude (pA)*** | 88.17 ± 8.15 | 9.23 – 2662.11 | 11.59 ± 0.13 | 7.02 -42.88 | |
| ***Rise time (ms)*** | 206.28 ± 12.28 | 21.33 – 1563.86 | 6.1 ± 0.11 | 1.5 - 16 | |
| ***Decay time (ms)*** | 753.99 ± 143.85 | 25 - 50596 | 24.42 ± 0.41 | 3.8 – 40.1 | |
| ***Area (pC)*** | 39.162 ± 3.453 | 0.635 – 724.807 | 0.151 ± 0.003 | 0.032 – 0.914 | |
| **Human** | | | | | |
| ***Parameter*** | **SIC** | | **EPSC** | | **p** |
|  | Average ± SEM | range | Average ± SEM | range |  |
| ***Amplitude (pA)*** | 51.58 ± 13.6 | 13 - 324 | 12.56 ± 0.35 | 6.7 – 38.6 | 0.13 |
| ***Rise time (ms)*** | 170.1 ± 68.18 | 42.9 - 1304 | 5.69 ± 0.28 | 0.4 – 15.6 | 0.25 |
| ***Decay time (ms)*** | 765.73 ± 461.65 | 39.94 - 9002 | 15.39 ± 0.72 | 1.3 -40.1 | 0.49 |
| ***Area (pC)*** | 101.89 ± 34.13 | 634.4 – 2007.36 | 125.08 ± 8.86 | 8.33 - 1041 | < 0.0001 |

Supplementary Table 1. Comparison of murine and human EPSC and SIC parameters. All parameters of SICs and EPSCs were statistically different (p < 0.0001). P values of the table are from comparison of human and murine SIC parameters.

|  | **aCSF** | **500 nM PPPA** | **5 µM ifenprodil** | **D-AP5** | **p** |
| --- | --- | --- | --- | --- | --- |
| ***Mouse SIC activity (pC/min) NMDA receptor subunit pharmacology*** | 28.32 ± 5.01 | 36.97 ± 10.79 | 5.21 ± 2.46 | 0 | aCSF vs. PPPA: 0.746  aCSF vs. ifenprodil:  0.046  PPPA vs. ifenprodil:  0.003 |
|  | **aCSF** | **0.1% DMSO** | **10 µM CNO** |  | **p** |
| ***Mouse SIC activity (pC/min) chemogenetic astrocyte activation in hM3D-expressing mice*** | 5.79 ± 2.81 | 6.22 ± 2.66 | 35.43 ± 11.1 |  | aCSF vs. CNO, DMSO vs. CNO:  0.011 |
| ***Mouse SIC activity (pC/min) chemogenetic astrocyte activation in mCherry-expressing mice*** | 0.82 ± 0.53 | 3.5 ± 1.88 | 3.5 ± 2.63 |  | aCSF vs. CNO, DMSO vs. CNO:  0.58 |
|  | **aCSF** |  | **ifenprodil** | **D-AP5** | **p** |
| ***Human SIC activity (pC/min) NMDA receptor subunit pharmacology*** | 41.93 ± 17.59 |  | 1.55 ± 1.13 | 0.71 ± 0.71 | aCSF vs. ifenprodil: 0.02  aCSF vs. D-AP5: 0.018  ifenprodil vs. D-AP5:  0.99 |
|  | **aCSF** | **100 µM WAY213613** |  |  | **p** |
| ***Human SIC activity (pC/min) glutamate uptake inhibition*** | .26 ± 1.52 | 74.35 ± 31.23 |  |  | 0.02 |

Supplementary Table 2. Pharmacological and chemogenetic characterization of murine and human SICs.

|  | **‘negative shift'** | **‘zero shift’** | **‘positive shift’** | **p** |
| --- | --- | --- | --- | --- |
| ***Chemogenetically induced SIC*** | 1.12 ± 0.7 | 1.96 ± 0.39 | 0.93 ± 0.08 | negative vs. zero: 0.0015  zero vs. positive: 0.0002 |
| ***Glutamate uncaging*** | 1.21 ± 0.12 | 1.19 ± 0.01 | 0.79 ± 0.04 | negative vs. positive: 0.038 |
| ***SIC as postsynaptic electrical signal*** | 1.12 ± 0.11 | 1.22 ± 0.14 | 0.88 ± 0.06 | positive vs. zero: 0.041 |

Supplementary Table 3. Changes in murine EPSC amplitudes by SICs with various timings. EPSC amplitudes were normalized on amplitudes before SIC occurrence.

|  | **Before the first SIC** | **After the first SIC** | **Normalized parameter (after/before)** | **p** |
| --- | --- | --- | --- | --- |
| ***sEPSC amplitude (pA)*** | 12.13 ± 1.1 | 15.1 ± 1.3 | 1.25 ± 0.07 | 0.05 |
| ***sEPSC frequency (Hz)*** | 0.08 ± 0.02 | 0.16 ± 0.05 | 1.97 ± 0.33 | 0.08 |

Supplementary Table 4. Changes in human sEPSC parameters after the appearance of the first SIC.

|  | **Young** | **Middle-aged** | **Old** | **Fitting parameters** | **p** |
| --- | --- | --- | --- | --- | --- |
| **Mouse** (n = 395 SICs from 62 animals) | | | | | |
| ***Individual SIC charge transfer (pC)*** | 39.1 ± 3.95 | 33.13 ± 4.5 | 40.27 ± 5.6 | r^2^ = -0.0024; slope: 5.3 fC/day |  |
| ***SIC activity (pC/min)*** | 36.34 ± 6.81 | 17.63 ± 5.39 | 12.16 ± 3.6 | single exponential: r^2^ = 0.21, τ = 29.33  linear: r^2^ = 0.206, slope: -58.4 fC/min/day | young vs. old: 0.0011 |
| **Human** (n = 29 SICs from 12 patients) | | | | | |
| ***Individual SIC charge transfer (pC)*** | - | 273.9 ± 74.1 | 15.9 ± 8.6 | r^2^ = 0.36; slope: -9203.1 fC/year |  |
| ***SIC activity (pC/min)*** |  | 136.6 ± 43.4 | 4.03 ± 1.42 | single exponential: r^2^ = 0.484, τ = 13.34; linear: r^2^ = 0.467, slope: -3389.4 fC/min/year | middle-aged vs. old:  < 0.0001 |

Supplementary Table 5. Age-related changes in murine and human SIC parameters. Mouse age categories: young:8-30 days old, middle-aged: 90-200 days old, old: above 1 year. Human age categories: middle-aged: 38-45 years old, old: 59-74 years old.

|  | **Young (8-30 d.)** | **Middle-aged (90-200d.)** | **Old (1+ yr.)** |
| --- | --- | --- | --- |
| ***Absolute change of EPSC frequency after SIC*** | 0.445 ± 0.059 | 0.367 ± 0.15 | 0.332 ± 0.1 |
| ***Absolute change of EPSC amplitude after SIC*** | 0.063 ± 0.01 | 0.062 ± 0.02 | 0.047 ± 0.015 |

Supplementary Table 6. Age-dependence in the capability of SICs for eliciting synaptic plasticity in mice. There was no significant difference between the datasets.

|  | **Middle-aged**  **(38-45 yrs.)** | **Old**  **(59-74 yrs.)** | **p, fitting parameters** |
| --- | --- | --- | --- |
| ***Absolute change of EPSC frequency after SIC*** | 2.59 ± 0.22 | 1.34 ± 0.32 | r^2^ = 0.634; slope: -0.057/year  p= 0.015 |
| ***Absolute change of EPSC amplitude after SIC*** | 1.18 ± 0.12 | 1.33 ± 0.08 | r^2^ = 0.11 and slope: 0.008/year  p= 0.16 |

Supplementary Table 7. Age-dependence in the capability of SICs for eliciting synaptic plasticity in humans.
